# Vision-Specific Tools for the Assessment of Health-Related Quality of Life (HR-QoL) in Children and Adolescents with Visual Impairment: A Scoping Review

**DOI:** 10.3390/ijerph21081009

**Published:** 2024-07-31

**Authors:** Tshubelela Sello Simon Magakwe, Rekha Hansraj, Zamadonda Nokuthula Xulu-Kasaba

**Affiliations:** Discipline of Optometry, University of KwaZulu Natal, Durban, KwaZulu Natal 4001, South Africa; hansrajr@ukzn.ac.za (R.H.); xulukasabaz@ukzn.ac.za (Z.N.X.-K.)

**Keywords:** vision-specific instrument, health-related quality of life, visual impairment, children, adolescents

## Abstract

Vision-related quality-of-life (QoL) measures offer a comprehensive evaluation of the impact of eye conditions and the effectiveness of treatment on important aspects of QoL. A substantial number of tools for assessing health-related quality of life (HR-QoL) in adults have been reviewed. However, despite the high prevalence of uncorrected refractive errors causing visual impairment (VI) in children, there is a notable lack of similar tools for this vulnerable population. This review aimed to systemically map evidence on the availability and use of vision-specific instruments for assessing HR-QoL in children and adolescents with VI. This review follows the Joanna Briggs Institute (JBI) guidelines (2020) and the framework by Arksey and O’Malley and Levac et al. (2010). We conducted systematic searches through databases PubMed, Science Direct, and Scopus and search platforms Web of Science and EBSCOhost to source reviews published in English from the date of their inception to December 2023. The findings are reported according to the Preferred Reporting Items for Systematic Reviews and Meta-Analyses extension for scoping reviews (PRISMA-ScR). We reviewed twenty tools, nine of which were developed for children in the United States and three of which were developed for children in developing countries; no tools specifically developed for children in Africa were found. In the reviewed papers, the tools were presented to children, parents, or proxies in an interview or questionnaire format. For most of the tools, reliability was assessed using internal consistency (n = 12) and test–retest reliability (n = 12). The most dominant measures of validity were construct (n = 16), content (n = 8), internal (n = 4), and criterion (n = 4). There appears to be a need for more tools developed for children in middle–low-income countries, especially for African children.

## 1. Introduction

Visual impairment (VI) poses a serious threat to the quality of life (QoL) of children, as 85% of their daily activities depend on vision [1]. Moreover, it was reported that most children with early-onset severe VI have delayed motor, language, emotional, social, and cognitive development [2]. The World Health Organization (WHO) estimates that 18.94 million children are visually impaired, of which 1.42 million children are blind [3]. They further state that childhood blindness accounts for 3.64% of the total population of blind people globally [3]. However, these data ignore the impact of blindness in childhood because it compares children with adults, while children live with blindness for a longer time [4]. Visual impairment is associated with a decreased QoL, which has been defined as one of the complex traits that includes visual functioning, patient’s symptoms, emotional well-being, and social relationships [5]. In children, VI decreases QoL in various ways, including causing difficulty in reading from the board in classrooms and reading books, restrictions on activities, limitations in mobility, and decreased chances of employment in the future [6]. Furthermore, children with VI show reduced engagement in social and pleasurable activities [5]. It has also been well established that VI and blindness are risk factors for both anxiety and depression [7,8].

In research and clinical care, progress in addressing visually impairing disorders is primarily measured and understood using objective visual function tests: for example, tests of visual acuity or the visual field, both of which can be insensitive to the presence of eye disease and therefore fail to capture visual disability completely [9]. An in-depth understanding of the impact of visually impairing disorders and treatment is required [6], including subjective complaints, which are challenging to measure and thus often receive less attention, particularly amongst children and adolescents, while they are of utmost clinical importance to guide progress in addressing visually impairing disorders and access to care rehabilitation [10]. The need for patient-reported information has led to the development of numerous questionnaires, often referred to as instruments or tools [6]. These tools have been reported to serve as reliable, relevant, and cost-effective methods to assess, to an extent, the degree of VI that children and adolescents experience in their daily activities [10]. Moreover, studies have shown that children can answer any health-related quality-of-life (HR-QOL) questionnaire reliably if their reading skills, cognitive capacity, and emotional development were considered during the development of those questionnaires [1,11].

Vision-related quality-of-life (QoL) measures offer a comprehensive evaluation of the impact of eye conditions and the effectiveness of treatment on other important aspects of QoL [10]. Despite the high prevalence of uncorrected refractive errors causing VI in children, a substantial number of tools for assessing health-related QoL in adults have been reviewed [9]. However, similar assessments for children and adolescents are lacking [1]. In addition, there exists a lack of clarity regarding the availability and use of vision-specific instruments for assessing HR-QoL in children and adolescents with VI, and the benefits and appropriateness of the currently available vision-specific instruments for use in resource-limited settings remain uncertain.

## 2. Objective

We conducted a scoping review of published peer-reviewed literature to (1) systematically map evidence on the availability and use of vision-specific instruments for assessing HR-QoL in children and adolescents with VI; (2) synthesize the findings of studies addressing HR-QoL in children and adolescents with VI; and, finally, (3) describe the psychometric properties of currently available vision-specific instruments for assessing HR-QoL in children and adolescents with VI.

## 3. Materials and Methods

The design and conduct of this review were guided by the scoping review framework suggested by Arksey and O’Malley (2005) and the guidelines by Levac et al. (2010) for methodological enhancement in conducting a scoping review project [12,13]. According to the framework suggested by Arksey and O’Malley (2005), there are five different stages in undertaking a scoping review: (1) defining the review question and developing criteria for including studies; (2) searching for studies addressing the review question; (3) selecting studies meeting the criteria for inclusion in the review; (4) charting the data from the studies meeting the criteria for inclusion; and (5) collating, summarizing, and reporting the results.

The review results are reported in accordance with the Preferred Reporting Items for Systematic Reviews and Meta-Analyses extension for scoping reviews (PRISMA-ScR) [14]; the completed checklist can be accessed on the Zenodo repository via the following link: DOI: 10.5281/zenodo.11537654 [15]. Before the study commenced, the review protocol was registered on the Open Science Framework at https://doi.org/10.17605/OSF.IO/9HW8C [16], and it is currently under review for publication in the *F1000Research* journal (manuscript ref # 151839).

### 3.1. The Review Question and Criteria for Including Studies

To determine the research question’s eligibility for a scoping review project, we applied the PCC (Population (or Participants), Concept, and Context) nomenclature framework recommended by the Joanna Briggs Institute (JBI) Manual for Evidence Synthesis: 2020 Edition [17], as illustrated in Table 1.

### 3.2. The Research Questions Addressed in This Scoping Review

The following research questions are addressed in this scoping review:

1. What evidence exists on the availability and usage of vision-specific tools for assessing health-related quality of life (HR-QoL) in children and adolescents with visual impairments?
2. What are the research outcomes of assessing HR-QoL in children and adolescents with visual impairments?
3. What are the psychometric properties of current vision-specific tools for assessing HR-QoL in children and adolescents with visual impairments?

The eligible studies were included only after two independent reviewers, T.S.S. and A.K., had thoroughly evaluated them and confirmed their eligibility based on the criteria outlined in the PCC framework.

### 3.3. Search Strategy for the Identification of Studies Addressing the Review Question

Systematic, comprehensive, and reproducible searches of reputable bibliographic databases and indexing services (and platforms), followed by other supplementary information sources, were used to find primary studies addressing the main review question, which were thereafter screened for their eligibility for inclusion in this review. T.S.S. (an optometrist by profession) and A.K. (a social worker by profession) performed all of the primary electronic and supplementary systematic searches using a pre-defined search strategy with the assistance of professional librarians based at the University of Free State and the University of KwaZulu-Natal.

The first author and a subject specialist co-developed the comprehensive search strategy. All authors reviewed the draft to ensure the correct use of indexing terminology and Medical Subject Headings (MeSH) descriptors before it was piloted on a subset of records from the PubMed database.

### 3.4. Electronic Search Sources

Various databases, such as PubMed, Science Direct, Scopus, Web of Science, and EBSCOhost, were systematically searched for peer-reviewed articles published in English between the date of their inception and December 2023. The EBSCOhost platform included databases such as Academic Search Complete, Health Source: Consumer Edition, Health Source: Nursing/Academic Edition, and Open Dissertation. The searches used both free text and controlled vocabulary terms (e.g., MeSH) to extract relevant articles.

To gather more resources, the first author performed a manual search by browsing through the “Related Articles” link to find additional studies. Only primary research studies were included, while systematic reviews and other formats of published literature review papers, whether peer-reviewed or not, were excluded. However, the reference lists of relevant reviews, preprints, conference abstract papers, and full-text articles were screened to find more primary studies. After completing the search, all of the citations found were uploaded into Mendeley version 2.76.0/2023.

### 3.5. Study Selection and Eligibility Screening

The process of selecting studies involved multiple steps and was conducted by two independent reviewers, T.S.S. and A.K., to reduce errors and bias following the Preferred Reporting Items for Systematic Reviews and Meta-Analysis extension for scoping reviews (PRISMA-ScR). The study selection process consisted of three screening stages: title screening, the screening of abstracts, and full-text article screening. Both reviewers independently screened the titles and abstracts of the references retrieved through the search strategies based on predetermined inclusion and exclusion criteria. Any discrepancies were resolved through discussion and, if necessary, referred to a third reviewer.

After completing the title and abstract screening process, the full-text articles were retrieved for studies that met the inclusion criteria or where there was uncertainty. These articles were then screened in greater depth to determine their eligibility for inclusion. T.S.S. and A.K. independently assessed the full texts of potentially relevant studies for eligibility. Any study that did not meet the inclusion criteria was excluded. Once the full-text screening stage was completed, the two reviewers conducted a secondary search of the reference lists of all included studies in order to identify any relevant articles that were missed during the initial database search.

### 3.6. Data Items and Data Charting Methods

After screening the full text of the articles, two independent reviewers, T.S.S. and S.S. (both optometrists by profession), performed the data extraction process using a standardized form or checklist to ensure a systematic data extraction process. The screening tools used can be found in the Supplementary File which can be access from Zenodo repository for free using this link DOI: 10.5281/zenodo.11537918 [23]. The first author extracted quantitative data, which was then checked by another reviewer. Any discrepancies were resolved by discussion, and a third reviewer was available if needed. Two reviewers worked separately to extract qualitative data such as the study aim, sample, type, and nature of the intervention/programme, theoretical approach, and methods used to collect data, and analytical processes. This was performed in duplicate to identify inter-rater errors and reduce data errors and bias. Various components of each vision-specific instrument were then extracted and summarized, including the frequency of its use in studies, age parameters, profiles of the respondents, the number of items assessed, scale, scoring system, domains assessed, validity, and reliability.

## 4. Results

**Figure 1:**
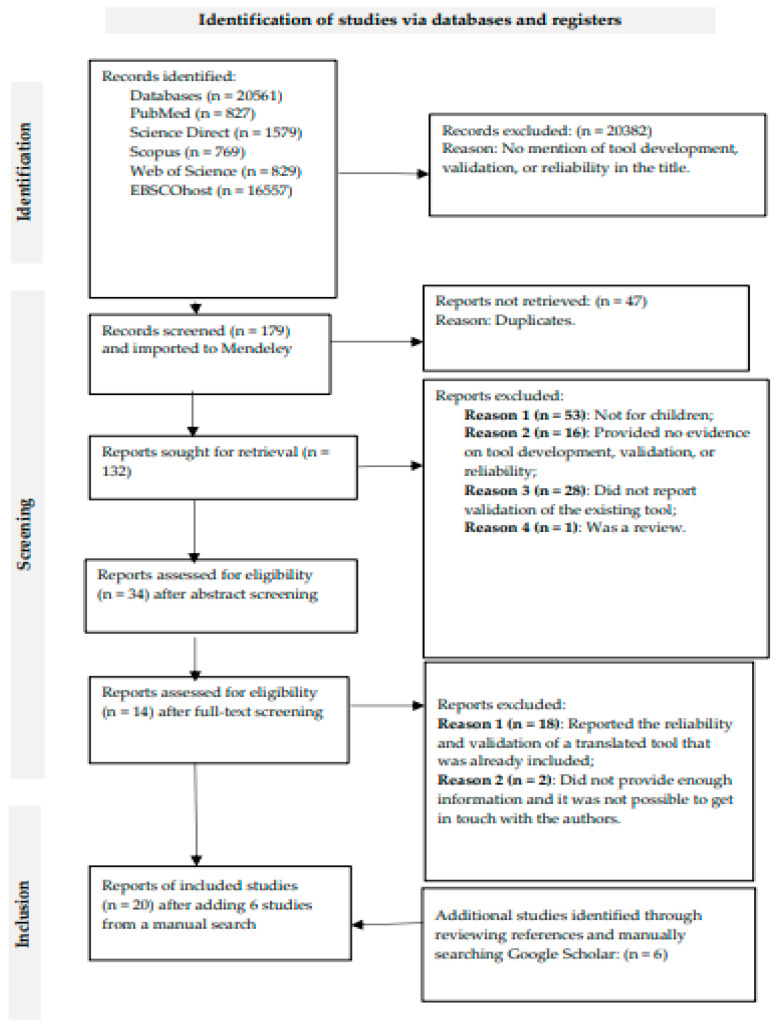
PRISMA 2020 flow diagram for systematic reviews, including searches of databases and registries.

A total of 20,561 resources were searched across various databases and platforms using the initial search strategy. After title screening, 20,382 resources were excluded as they did not provide any information regarding the development, reliability, validity, or translation of the tool. Out of the 179 resources imported to Mendeley, 132 resources remained for abstract screening after removing duplicates. Two reviewers, T.S.S. and A.K., agreed on including 32 resources for full-text screening and excluded 98 resources for reasons such as not reporting on children, reporting on the validation or reliability of already-included tools, or being duplicates. After full-text screening, T.S.S. and A.K. excluded 20 resources as they reported validations of tools but had no evidence of tool development, or were outside the definition of children and adolescents. Hence, a total of 14 resources met the inclusion criteria. Additionally, 6 resources were retrieved from a manual search, bringing the total number of resources up to 20. A PRISMA flowchart of the selection process is shown in Figure 1, and the results were analyzed and synthesized based on the three aforementioned themes.

### 4.1. Characteristics of Sources of Evidence

All eighteen peer-reviewed journals and two peer-reviewed theses reported on developing, validating, and testing the reliability of vision-related QoL and/or functional vision assessment tools for children and adolescents. According to Table 2, most of these tools were developed in the United States of America (n = 9), while Australia, China, Saudi Arabia, and Italy developed only a single tool each. African countries were not targeted for the development of these tools. Even though we were focused on tools covering children 18 years and younger, we decided to include one tool that covered the age group of between 10 and 20 years because it was the only study that focused on refractive error tool development (Student Refractive Error and Eyeglasses Questionnaire (SREEQ)). A total of 14 studies used questionnaires or surveys as the most common method of data collection, while 4 studies combined questionnaires with interviews. Almost all of the studies (n = 18) used the English language to develop the tool (from 1999 to 2020). However, at least four tools have been translated into more than one language in addition to English. 

Table 2 shows that eight of the studies did not have any race limitations, while two of them did not report on their targeted race. The highest number of respondents were children (n = 12), followed by children in conjunction with their parents (n = 6), and then proxy respondents (which were either parents alone or parents and caregivers) (n = 2). Most of the studies measured vision-related QoL (n = 10), followed by functional vision (n = 6), and health-related QoL (n = 2). The tools used in these studies covered various eye conditions, including VI (n = 9), amblyopia (n = 3), refractive error (n = 2), uveitis, allergic conjunctivitis, convergence insufficiency (n = 1), and intermittent exotropia (n = 1). Additionally, two of the studies were not limited to any specific eye conditions. The most used mode of administration in this review was noted to be self-reporting (n = 13); the interview format was the least used (n = 3), and 4 studies utilized both modes.

The majority of the tools were created through the following methods: extracting information from a literature review (n = 9), conducting focus group discussions with children and/or experts (n = 7), consulting with experts through interviews (n = 7), interviewing children and their parents (n = 7), and constructing new tools by using existing ones as a basis (n = 2), as indicated in Table 3 below. The 5-point task difficulty scale was the commonly used rating scale (n = 7), followed by the frequency rating scale, and the level-of-happiness scale, which was utilized in only a single paper. In all of the tools, the number of domains measured ranged from 1, being the least, to 12, with the number of items ranging from 11 to 90, as shown in Table 3.

### 4.2. Results of Individual Sources of Evidence

VRQOL-JIA

The Vision-Related Quality-of-Life Instrument for use in Juvenile Idiopathic Arthritis-associated Uveitis (VRQOL-JIA) is a QoL assessment tool designed specifically to assess the impact of vision-related issues on children aged 8–18 years living in the USA who suffer from juvenile idiopathic arthritis-associated uveitis [17]. This tool was developed through focus group discussions with experts and children and a thorough literature review. It measures both visual function and QoL related to vision. The questionnaire is completed by either the child with uveitis themselves or their parents, using a six-point vision severity scale and a five-point task difficulty scale. The tool consists of 12 domains, with 23 items for children aged 8–15 years and 26 items for individuals aged 16–18 years. The reliability of this tool was tested using test–retest reliability and the Cronbach alpha coefficient, while its validity was examined through both constructive and criterion validity methods.

CVAQC

The Cardiff Visual Ability Questionnaire for Children (CVAQC) [21] is a purpose-built tool for evaluating the visual acuity of children between the ages of 5 and 18 years residing in the UK. This instrument was carefully developed through focus group discussions with children, ensuring its relevance and comprehensibility. It is presented to children in an interview format, utilizing a six-point scale to ascertain the level of task difficulty. With seven domains and 25 items, this tool comprehensively covers all aspects of visual ability. To test its reliability, the tool underwent a test–retest analysis using intraclass correlation (ICC), and the constructive validity method was implemented for validation purposes. 

PedEyeQ

The Pediatric Eye Questionnaires (PedEyeQ) [22] present a valuable resource for assessing the QoL and functional vision of children up to 18 years of age in the USA. Developed through a thorough literature review, this tool is completed by either children, parents, or proxies using a four-point frequency scale. Divided into five domains and consisting of 40 items, the reliability of this tool was established through internal consistency using the Cronbach alpha test, and its validity was confirmed through constructive validity analysis.

CVFQ

The Children’s Visual Function Questionnaire (CVFQ) [24] was created to evaluate the impact of visual impairments on the QoL of children up to 7 years of age residing in the USA. Experts were consulted in developing this questionnaire, which is completed by parents or proxies using a variety of five-point scales to rate visual status, agreement, task difficulty, and frequency. This tool consists of six domains, with 35 items for children under 3 years and 40 items for those aged 3–7. Its reliability was assessed through test–retest reliability and Cronbach alpha coefficient analyses for internal consistency, while constructive validity was used to validate this tool.

LVP-FVQ I

The LV Prasad Functional Vision Questionnaire (LVP-FVQ I) [25] is a tool that was designed to evaluate functional vision among Indian children between the ages of 8 and 18 years. The development process involved a thorough review of the existing literature, as well as focus group discussions with both children and experts. This questionnaire is presented to children in an interview format, utilizing a five-point task difficulty scale and consisting of four domains with a total of 20 items. To ensure its reliability, test–retest methods were utilized, while content, constructive, and criterion validity analyses were employed for validation purposes.

LVP-FVQ II

The LV Prasad Functional Vision Questionnaire Second Version (LVP-FVQ II) [26] is a second-version tool designed to evaluate functional vision among children aged 8–16 years living in India. The development process involved an extraction of knowledge from the literature via a literature review and focus group discussion with experts. This questionnaire is presented to children in an interview format, like the first version, and it utilizes a three-point task difficulty scale as well as a three-point global rating scale to compare respondents to their peers. The LVP-FVQ II tool consists of four domains and 23 items. To test its reliability, test–retest reliability using interclass correlation and internal consistency via the Cronbach alpha coefficient were measured. Moreover, it was validated through assessments of its content and construct. The LVP-FVQ II tool covers more domains and includes items related to mobility, which the first version did not have.

VRQOL-YC

The Vision-Related QoL in Young Children (VRQOL-YC) tool [27] is a tool that has been specifically designed to measure the visual-related QoL of children up to 7 years of age, who are residents of the USA. It was developed through a careful analysis of clinical expertise and the literature. The questionnaire, comprising 61 items, is completed by a proxy using a five-point QoL rating scale and covers six core domains. This tool’s reliability was assessed through the Cronbach alpha coefficient, while its validity was tested using both content and constructive validity methods.

QUICK

The Health-Related Quality of Life in Children with Vernal Keratoconjunctivitis Questionnaire (QUICK) [28] is a tool that evaluates the QoL of children aged 4–12 years who are living in Italy and who have been diagnosed with vernal keratoconjunctivitis. This questionnaire was developed through an extensive literature review, consultations with researchers, and interviews with children and their parents. It consists of 16 items grouped into seven domains, and children complete the questionnaire using a three-point frequency scale. The reliability of the QUICK was evaluated using the Cronbach alpha coefficient, and its validity was confirmed through criterion and constructive validity testing.

FVQ-CYP

The Functional Vision Questionnaire for Children and Young People with Visual Impairment (FVQ-CYP) [29] is a tool that was designed to evaluate the functional vision of children aged 10–15 years residing in the United Kingdom. This questionnaire was developed through interviews with children and experts. It is completed by the children themselves using a five-point scale that rates task performance. This tool consists of four domains and 36 items. There are no reports available on how its reliability has been assessed. However, its constructive validity was assessed using the personal correlation coefficient (PCC).

VRQOL-CID

The Vision-Related Quality of Life (VRQOL-CID) [30] tool is designed for Chinese children aged 8–18 years who have intellectual disability (ID). This tool helps in assessing vision-related quality of life and has 16 domains and 90 items. The questionnaire uses a four-point scale that rates the level of happiness scale and is completed by children, parents, and proxies. This tool was developed after an extensive literature review and focus group discussions with parents and caregivers. Its reliability was evaluated using test–retest reliability and the Cronbach alpha coefficient, while its validity was assessed via constructive and internal validity analyses. 

CVLS

The Children’s Vision for Living Scale (CVLS) [31] is a tool designed to measure QoL related to vision in children aged 5–12 years who live in Saudi Arabia. The development of this tool involved an extensive literature review and consultation with experts in children’s health, parents, and children. Children with amblyopia complete this questionnaire, which comprises six domains and 21 items. The scale uses a five-point frequency rating and a five-point severity rating. The reliability of this tool was assessed using the Cronbach alpha coefficient, personal separation, and item separation, while its validity was assessed via construct and internal validity analyses.

IVI_C

The Impact of Vision Impairment on Children (IVI_C) questionnaire [32] is a tool designed to evaluate the vision-related QoL of children aged 8–18 years who are living with VI in Australia. This instrument was created through focus group discussions with children, parents, and teachers. It is available in both questionnaire and interview formats, with both being completed by the children themselves. This tool consists of 24 items rated on a five-point frequency scale, but the specific domains that it assesses have not been reported. The reliability of this tool was established through test–retest reliability, equivalence (inter-observer reliability), and internal reliability measures, while its validity was determined through content and construct validity analyses. 

PRO-FV

The Patient-Reported Outcome Measure of Functional Vision (PRO-FV) tool [33] was developed to assess functional vision among children and young people aged 8 to 18 years with VI living in the United Kingdom. This tool was developed from modified items of the FVQ-CYP tool and consists of both a questionnaire and an interview format. Its respondents are children, and the tool consists of two domains assessed through 41 items, using a four-point task difficulty scale. The Pearson correlation coefficient was used to assess its test–retest reliability, and constructive validity was used to measure its validity.

CHVI-VFQ

The Visual Function Questionnaire for Children with Visual Impairment (CHVI-VFQ) [34] is a tool that is used to measure the visual function of children in India aged 5–15 years. This tool was created by combining already-validated tools, namely LVP-FVQ I and II, and by conducting consultation workshops with experts. This questionnaire consists of 43 items and four domains. The questionnaire is completed by children and proxies, using a four-point scale rating the condition’s severity and a three-point scale rating task difficulty. To test for its reliability, the Cronbach alpha coefficient was employed, and the tool was internally validated as a method of validation. 

ATI

The Amblyopia Treatment Index (ATI) [35] is a tool designed to evaluate the effectiveness of amblyopia treatment among children between the ages of 3 and 13 years who live with the condition in the USA. The ATI was developed based on an extensive review of the literature and clinical experience. It comprises six domains, with 20 items for children aged 3–6 years and 19 items for children aged 7–13 years. This questionnaire is completed by both children and proxies, using a five-point agreement scale. The test–retest reliability of this tool was evaluated using the Pearson correlation coefficient; its internal consistency was assessed to measure its reliability; and it was validated using internal validity analysis.

CAT-QoL

The Children’s Amblyopia Treatment Quality of Life Questionnaire (CAT-QoL) [36] is a questionnaire designed to gauge the QoL of children aged 5–7 years with amblyopia who live in the United Kingdom. This tool was developed through interviews with children and consists of 11 items. It uses a five-point scale that rates symptom severity and can be completed by the children themselves. However, there is no report on the number of domains it assesses. To assess its reliability, this tool underwent an evaluation of its Cronbach alpha coefficient and equivalence, while internal validity was used to assess its validity.

CISS

The Convergence Insufficiency Symptom Survey (CISS) [37] is a tool used to assess the symptom severity of children aged 9–18 years diagnosed with convergence insufficiency residing in the USA. This tool was developed through consultations for investigating the perspectives of researchers and children and has one domain assessed through 15 items. This tool is presented to children in an interview format and/or a questionnaire format using a five-point scale rating symptom severity. To assess its reliability, this tool underwent a test–retest reliability analysis, and for validation, constructive and internal validity analyses were employed.

IXTQ

The Intermittent Exotropia Questionnaire (IXTQ) [38] is a tool designed to measure the QoL of children aged 5–17 years who have been diagnosed with intermittent exotropia and live in the USA. The development of this tool involved interviews with children and their parents. It comprises three domains and a total of 12 items for children, 12 items for proxies, and 17 items for parents. This questionnaire uses five-point and three-point agreement scales and is completed by children, proxies, and parents. The reliability of this questionnaire was evaluated through a measurement of its Cronbach alpha coefficient, and its validation included a discriminant validity analysis.

PREP2

The Pediatric Refractive Error Profile (PREP2) [39] is a tool designed to evaluate the vision-related function of children aged 8–18 years who have been diagnosed with refractive error and who reside in the USA. The development of this tool involved consultations with children and their parents, and it includes ten domains assessed through 56 items. The questionnaire is filled out by children using a five-point agreement scale. To assess the reliability of this tool, its test–retest reliability and internal consistency were measured, while constructive validity was used to validate this tool.

SREEQ

The Student Refractive Error and Eyeglasses Questionnaire (SREEQ) [40] is a tool designed to measure the vision-related QoL of children aged 10–20 years who wear spectacles due to having refractive errors and who reside in the USA. This tool was developed by a team of researchers who utilized their expertise in the field, and it comprises two domains evaluated through a total of 23 items. The questionnaire is filled out by the children themselves using a three-point frequency scale. The reliability of this tool was assessed using the Cronbach alpha coefficient and Pearson reliability methods, while its validity was examined through content and discriminant validity methods.

### 4.3. Psychometric Properties of the Tools

The reliability of a study is determined by its ability to consistently produce the same results when repeated [41]. This can be assessed by measuring the stability of a tool, test–retest reliability (reproducibility), and internal consistency. Test–retest, or reproducibility, refers to the extent to which scores remain consistent over time when no changes are expected [42]. It evaluates if stable patients score similarly on a certain measure between two assessments in a relatively short period of time. It is recommended that the intraclass correlation coefficients be higher than 0.60 in stable patients over a two-week period [43]. Test–retest reliability was evaluated for the following instruments: VRQOL-JIA, CVAQC, CVFQ, LVP-FVQ I, LVP-FVQ II, VRQOL-CID, IVI_I, PROFV-CY, ATI, CISS, and PREP2. Internal consistency refers to the degree to which all items measure the same underlying construct and is typically evaluated using the Cronbach’s coefficient alpha formula [44]. A Cronbach alpha value of 0.70 or higher is generally considered indicative of satisfactory reliability [45]. Among the questionnaires used to assess internal consistency, the following were included: VRQOL-JIA, PedEyeQ, CVFQ, VRQOL-YC, QUICK, VRQOL-CID, CVLS, PROFV-CY, CHVI-VFQ, ATI, CAT-QoL, IXTQ, PREP2, and SREEQ. Notably, the FVQ-CYP tool did not provide any information regarding reliability, and seven tools were utilized through multiple methods for testing their reliability. It is important to note that while an instrument may demonstrate reliability, this does not guarantee accurate measurement of the intended construct; therefore, validating the tool is required.

Validity pertains to the extent to which an instrument accurately measures what it intendeds to measure [44]. This can be evaluated through content, construct, criterion, internal, and discriminant validity analyses. Content validity assesses how well an instrument captures crucial elements related to patients and the specific disease being studied [46]. In essence, this determines whether the instrument effectively reflects the concerns and perspectives of the patients. Tools that underwent this form of validation were CVAQC, LVP-FVQ I, VRQOL-YC, CVLS, and IVI_C. Construct validity evaluates how well a test assesses the concept for which it was designed [47]. Instruments validated through this validation method include VRQOL-JIA, PedEyeQ, CVFQ, LVP-FVQ I, VRQOL-YC, QUICK, FVQ-CYP, VRQOL-CID, CVLS, IVI_C, PROFV-CY, CISS, IXTQ, PREP2, and SREEQ. Criterion validity measures the extent to which there is a relationship between a given test score and performance on another relevant measure [48,49]. There are two forms of criterion validation: predictive validity and concurrent criterion validity. This form of validation was utilized in the development of VRQOL-JIA, LVP-FVQ II, and QUICK. The concept of internal validity refers to the degree of confidence that the observed causal relationship in a study is not influenced by other extraneous factors or variables [42,45]. This form of validation was employed during the development of the following tools: VRQOL-CID, CHVI-VFQ, ATI, CAT-QoL, and CISS.

## 5. Discussion

Health services are increasingly emphasizing the importance of understanding how patients perceive the status of their vision [50]. In the field of ophthalmic research, there is growing recognition of the significance of measuring QoL as a supplementary indicator [48]. Historically, eye care professionals have predominantly relied on clinical examinations and input from parents, proxies, or teachers to assess the impact of vision issues on children, acknowledging the challenge in relying on children to express their vision problems [21,24,51]. However, there are concerns about whether assessments like visual acuity, contrast sensitivity, and visual field assessments accurately capture the child’s perspective [24]. Quality-of-life assessment tools have been identified as reliable, relevant, and cost-effective means of evaluating the impact of vision problems on school children to some extent [52]. These self-reported tools are preferable to proxy assessments. Additionally, research indicates that children can reliably respond to health-related quality-of-life (HR-QOL) questionnaires if their reading skills, cognitive capacity, and emotional development have been considered in the questionnaire’s design [53,54]. While the National Eye Institute Visual Function Questionnaire (NEI-VFQ) has been successfully used to measure QoL in individuals with visual impairment [55], its usefulness in assessing children is limited due to the inclusion of items not applicable to young children, such as those related to driving, and the exclusion of items like seeing the blackboard, writing in a straight line, and color perception, which are vital for growth and development during childhood.

While a few validated instruments have been developed specifically for use in children, over 80% of these were developed based on children in developed countries [19,20,21,24,25,29,30,31,32,33,35,36,37,38,39,40]. This makes some aspects, such as driving and reading traffic signs, irrelevant for children in developing countries. It also means that they overlook items familiar to children in developing countries, such as walking home through unpaved roads, playing soccer on uneven gravel playgrounds, and learning under poor lighting. Additionally, expected differences in socioeconomic status and cultural disparities between developed and developing countries, like those in Africa, make the effectiveness of these instruments somewhat unreliable when used with African children. Furthermore, most of these instruments were developed with a focus on developing countries, where children’s hobbies may often include reading and video games, in contrast to the “outside-of-school” activities of their counterparts in most rural parts of Africa, which may include tending to domestic animals and crops, playing games outside, and fetching water from the river. Even though the highest prevalence of children living with VI is found within developing countries [56], only three instruments have been designed based on children in developing countries, namely the LV Prasad Functional Vision Questionnaire (LVP-FVQ I), LVP-FVQ II, and CHVI-VFQ [25,26,34]. However, these instruments were designed specifically for children living in India, and thus they might be culturally irrelevant to children in most parts of Africa.

This review highlights the lack of tools specifically designed to measure the quality of life related to vision of children from developing countries. The information from this review, particularly the methodologies used in developing the tools, will be valuable in creating a similar tool for children in Africa.

## 6. Limitations

This review focused only on studies that were published in English, which means that it might have overlooked valuable information from studies published in other languages.

## 7. Conclusions

This study has identified 20 available tools for measuring vision-related quality of life that have been designed and validated with a focus on developed countries. There appears to be a need for more tools developed in English for children in middle–low-income countries, especially for African children.

## Figures and Tables

**Table 1:** PCC framework for determining the review question and criteria for including studies.

Types of Participants
The participants that were included in this review were children and adolescents 18 years and younger living with visual impairment (VI). Visual impairment is defined as having a visual acuity worse than 6/12 that cannot be corrected with spectacles, contact lenses, medication, or surgery [18].
Concept
Vision-specific instruments used to assess the HR-QOL of children and adolescents living with VI were identified and reviewed. The instruments utilized in these studies were those developed and published in English. Components of the questionnaires assessed included frequency of use, age parameters, respondents, and domains assessed. “Respondent” refers to a person who completes the questionnaire. For younger children, the parent may act as a surrogate respondent or a structured interview may be conducted with a trained interviewer. The questionnaire format was stratified into the number of items, scale, scoring system, and reported time to completion. The number of items refers to the number of questions present in the questionnaire. In addition, HR-QoL instruments’ psychometric assessments were addressed according to validity and reliability. The assessment of psychometric testing was based on the article by Solans et al. [19]. Validity was divided into three components: construct validity, content validity, and criterion validity. Construct validity ensures that the instrument measures the intended domain and not other related variables [20]. Content validity refers to the appropriateness of the items used to measure the construct of interest [21]. Criterion validity refers to how well the instrument compares to an external “gold standard” marker [20]. Reliability refers to the consistency and reproducibility of results obtained from an instrument [21]. This is determined by the instrument’s test–retest reliability and internal consistency. A value of 0.7 or higher is considered acceptable according to Cronbach’s alpha [22].
Context
The context of this review will be open: studies from any setting will be considered because the quality-of-life questionnaire can be used in any setting, from primary healthcare to specialized psychological care.

**Table 2:** List of tools used for assessing vision-related quality of life or visual function in children and adolescents.

Instrument	Source	Country	Age of Respondents, in Years	Language	Ethnicity of Respondents	Concepts Measured	Ocular Condition Studied	Respondents	Mode of Administration
(VRQOL-JIA)	[17] A1	USA	8–18	English	Various	Vision-related QoL and/or visual function	Uveitis associated with juvenile idiopathic arthritis	Children, Parents	Self-reporting
(CVAQC)	[21]	UK	5–18	English, Chinese, Turkish	White	Visual ability	Visual impairment	Children	Interviews
(PedEyeQ)	[22]	USA	0–18	English	Various	Eye-related QoL and functional vision	Any eye condition	Children, Proxies, Parents	Self-reporting
(CVFQ)	[24]	USA	0–7	English, German	Various	Vision-related QoL	Any eye condition	Parents, Proxies	Self-reporting
(LVP-FVQ I)	[25]	India	8–18	English, Hindi, Telugu	Indian	Functional vision	Visual impairment	Children	Interviews
(LVP-FVQ II)	[26]	India	8–16	English, Hindi, Telugu	Indian	Functional vision	Visual impairment	Children	Interviews
(VRQOL-YC)	[27]	USA	0–7	English	Various	Vision-related QoL	Visual impairment	Proxies	Self-reporting
(QUICK)	[28]	Italy	4–12	Italian	NR	Disease-specific QoL	Allergic conjunctivitis	Children	Self-reporting
(FVQ-CYP)	[29]	UK	10–15	English	White	Functional vision	Visual impairment	Children	Self-reporting
(VRQOL-CID)	[30]	China	8–18	Chinese	Chinese	Vision-related QoL	Visual impairment	Children, Parents, Proxies	Self-reporting
(CVLS)	[31]	Saudi Arabia	5–12	English	Arabian	Vision-related QoL	Amblyopia	Children	Self-reporting
(IVI_C)	[32]	Australia	8–18	English	Australian	Vision-related QoL	Visual impairment	Children	Self-reporting, Interviews
(PROFV-CY)	[33]	UK	8–18	English	White	Functional vision	Visual impairment	Children	Interviews, Self-reporting
(CHVI-VFQ)	[34]	India	5–15	English, Hindi, Telugu	Indian	Visual function	Visual impairment	Children, Proxies	Self-reporting
(ATI)	[35]	USA	3–13	English	Various	Impact of amblyopia treatment	Amblyopia	Children, Proxies	Self-reporting
(CAT-QoL)	[36]	UK	5–7	English	Various	QoL	Amblyopia	Children	Interviews, Self-reporting
(CISS)	[37]	USA	9–18	English	NR	Symptoms	Convergence insufficiency	Children	Self-reporting, Interviews
(IXTQ)	[38]	USA	5–17	English	NR	Health-related QoL	Intermittent exotropia	Children, Proxies, Parents	Self-reporting
(PREP2)	[39]	USA	8–18	English	Various	Vision-related function	Refractive error	Children	Self-reporting
(SREEQ)	[40]	USA	10–20	English	Various	Vision-related QoL	Refractive error	Children	Self-reporting

(VRQOL-JIA) = Vision-Related Quality-of-Life Instrument for use in Juvenile Idiopathic Arthritis-associated Uveitis, (CVAQC) = Cardiff Visual Ability Questionnaire for Children, (PedEyeQ) = Pediatric Eye Questionnaires, (CVFQ) = Children’s Visual Function Questionnaire, (LVP-FVQ I) = LV Prasad Functional Vision Questionnaire, (LVP-FVQ II) = LV Prasad Functional Vision Questionnaire, Second Version, (VRQOL-YC) = Vision-Related Quality of Life in Young Children, (QUICK) = Health-Related Quality of Life in Children with Vernal Keratoconjunctivitis Questionnaire, (FVQ-CYP) = Functional Vision Questionnaire for Children and Young People with Visual Impairment, (VRQOL-CID) = Vision-Related and Subjective Quality of Life in Children with Intellectual Disability, (CVLS) = Children’s Vision for Living Scale, (IVI_C) = Impact of Vision Impairment on Children, (PROFV-CY) = Patient-Reported Outcome Measure of Functional Vision for Children and Young People aged 8 to 18 years with Visual Impairment, (CHVI-VFQ) = Vision Function Questionnaire for Children with Visual Impairment, (ATI) = Amblyopia Treatment Index, (CAT-QoL) = Children’s Amblyopia Treatment Quality of Life Questionnaire, (CISS) = Convergence Insufficiency Symptom Survey, (IXTQ) = Intermittent Exotropia Questionnaire, (PREP2) = Pediatric Refractive Error Profile, (SREEQ) = Student Refractive Error and Eyeglasses Questionnaire, NR = not reported, USA = United States of America, UK = United Kingdom.

**Table 3:** Methods used for the development, domains, rating, reliability, and validation of the tools.

Tool	Sources Used to Generate Items	Rating Scales	List of Domains (N=)	No. of Items	Reliability Testing	Validity Testing
(VRQOL-JIA)	Focus group discussions with experts and children; Knowledge extracted from the literature	6-point scale assessing the severity of visual impairment; 5-point Likert scale assessing the level of task difficulty.	Distance vision, near vision, color, night vision, functionality, photosensitivity, optional driving, global vision, physical functioning, emotional functioning, social functioning, and school functioning (12).	49	Test–retest reliability; Internal consistency	Content validity; Constructive validity; Criterion validity
(CVAQC)	Focus group discussions with children	6-point scale assessing the level of task difficulty.	Educational, near vision, distance vision, getting around, social interaction, entertainment, and sport (7).	25	Test–retest reliability	Content validity
(PedEyeQ)	Knowledge extracted from the literature	4-point frequency scale.	Distance vision, near vision, mental distress, social life, and psychological health (5).	40	Internal consistency	Constructive validity
(CVFQ)	Consultation with experts	5-point scale assessing visual status; 5-point agreement scale; 5-point task difficulty scale; 5-point frequency scale.	General health, general vision, competence, personality, familial impact, and treatment (6).	75	Test–retest reliability; Internal consistency	Constructive validity
(LVP-FVQ I)	Knowledge extracted from the literature; Focus group discussions with experts and children	5-point Likert scale assessing the level of task difficulty; Yes/No options were available, where “No” meant there were no difficulties in performing a task.	Distance vision, near vision, color vision, and visual field (4).	20	Test–retest reliability	Content validity; Constructive validity; Criterion validity
(LVP-FVQ II)	Knowledge extracted from the literature; Focus group discussions with experts.	3-point Likert scale assessing difficulty; 3-point scale assessing global rating as compared to peers.	Activities of daily living, academic life, and leisure activities (4).	23	Test-retest reliability	Criterion validity
(VRQOL-YC)	Clinical expertise in the literature	5-point scale assessing quality of life.	General health, general vision, competence, personality, familial impact, and treatment (6).	61	Internal consistency	Content validity; Constructive validity
(QUICK)	Knowledge extracted from the literature; Consultations with researchers; Interviews with children and parents	3-point frequency scale.	Physical well-being, emotional well-being, self-esteem, family, friends, school life, and disease (7).	16	Internal consistency	Criterion validity; Constructive validity
(FVQ-CYP)	Interviews with children and experts	5-point Likert scale assessing task performance.	Functioning, home life, school life, and leisure (4).	36	NR	Constructive validity; Content validity
(VRQOL-CID)	Knowledge extracted from the literature; Focus group discussions with parents and caregivers	4-point Likert scale rating the level of happiness; Yes/No options were also used.	School activities, health, social life, leisure activities, family life, academic performance, gross motor activities, fine motor activities, object discrimination, distance vision, treatment, general health, general vision, competence, personality, and familial impact (16).	90	Test–retest reliability; Internal consistency	Construct validity; Internal validity
(CVLS)	Knowledge extracted from the literature; Consultations with pediatrics experts, parents, and children	5-point Likert scale rating frequency and severity.	Mood, self-esteem, social relations, functional vision, visio-motor skills, and academic performance (6).	21	Internal consistency	Content validity; Constructive validity
(IVI_C)	Focus group discussions with children, parents, and teachers	5-point frequency scale.	NR	24	Test–retest reliability; Internal reliability	Content validity; Construct validity
(PROFV-CY)	An already-available tool, namely the FVQ-CYP tool	4-point scale assessing task difficulty.	Restrictions at home, school and leisure activities, and limitations on levels of functioning, mobility, and communication (2).	41	Test–retest reliability; Internal consistency	Constructive validity
(CHVI-VFQ)	Already-available tools, namely the LVP FVQ I and LVP FVQ II tools; Consultations and workshops with experts	4-point scale assessing the severity of the condition; 3-point scale assessing the level of difficulty in performing tasks.	Mobility, education, daily routine, and psychosocial well-being (4).	43	Internal consistency	Internal validity
(ATI)	Knowledge extracted from the literature; Clinical experience	5-point agreement scale.	Adverse effects, treatment compliance, social stigma, and functioning at near distance (6).	39	Test–retest reliability; Internal consistency	Internal validity
(CAT-QoL)	Interviews with children	5-point scale assessing severity.	NR	11	Internal consistency	Internal validity
(CISS)	Perspectives of/consultations with researchers and children	5-point scale assessing symptom severity.	Symptom severity (1).	15	Test–retest reliability	Constructive validity; Internal validity
(IXTQ)	Interviews with children and parents	5-point agreement scale; 3-point agreement scale	Functional vision, psychosocial well-being, and surgery (3).	41	Internal consistency	Constructive validity
(PREP2)	Consultations with children and parents	5-point agreement scale.	Overall vision, near vision, distance vision, symptoms, appearance, satisfaction, activities, academics, handling, and peer perception (10).	56	Test–retest reliability; Internal consistency	Constructive validity
(SREEQ)	Researcher’s teams and their expertise	3-point frequency scale.	Impact of uncorrected and corrected refractive error and vision-related quality of life (2).	23	Internal consistence	Content validity; Constructive validity

(VRQOL-JIA) = Vision-Related Quality-of-Life Instrument for use in Juvenile Idiopathic Arthritis-associated Uveitis, (CVAQC) = Cardiff Visual Ability Questionnaire for Children, (PedEyeQ) = Pediatric Eye Questionnaires, (CVFQ) = Children’s Visual Function Questionnaire, (LVP-FVQ I) = LV Prasad Functional Vision Questionnaire, (LVP-FVQ II) = LV Prasad Functional Vision Questionnaire Second Version, (VRQOL-YC) = Vision-Related Quality of Life in Young Children, (QUICK) = Health-Related Quality of Life in Children with Vernal Keratoconjunctivitis Questionnaire, (FVQ-CYP) = Functional Vision Questionnaire for Children and Young People with Visual Impairment, (VRQOL-CID) = Vision-Related and Subjective Quality of Life in Children with Intellectual Disability, (CVLS) = Children’s Vision for Living Scale, (IVI_C) = Impact of Vision Impairment on Children, (PROFV-CY) = Patient-Reported Outcome Measure of Functional Vision for Children and Young People aged 8 to 18 years with Visual Impairment, (CHVI-VFQ) = Vision Function Questionnaire for Children with Visual Impairment, (ATI) = Amblyopia Treatment Index, (CAT-QoL) = Children’s Amblyopia Treatment Quality of Life Questionnaire, (CISS) = Convergence Insufficiency Symptom Survey, (IXTQ) = Intermittent Exotropia Questionnaire, (PREP2) = Pediatric Refractive Error Profile, (SREEQ) = Student Refractive Error and Eyeglasses Questionnaire, NR = not reported.

## Data Availability

Protocol data for this review have been published by the Open Science Framework: https://doi.org/10.17605/OSF.IO/9HW8C [16]. The PRISMA ScR checklist has been completed and deposited online at the Zenodo repository: DOI: 10.5281/zenodo.11537654 [15]. The Supplementary Materials and extra data associated with this review have been deposited online at the Zenodo repository: DOI: 10.5281/zenodo.11537918 [23].

## Supplementary Materials

The supporting information can be downloaded at https://www.mdpi.com/article/10.3390/ijerph21081009/s1. The list of appendices has been deposited by the Zenodo repository as a supplementary document entitled “Supplementary Documents for Vision-specific Tools for the Assessment of health-related Quality of Life (HR-QoL) in Children and Adolescents with Visual Impairment: A Scoping Review”, and can be accessed via this link: DOI: 10.5281/zenodo.11537918 [23].
