# Supplementary material for: Vision-Specific Tools for the Assessment of Health-Related Quality of Life (HR-QoL) in Children and Adolescents with Visual Impairment: A Scoping Review
Source: Int J Environ Res Public Health. 2024 Jul 31;21(8):1009. doi: 10.3390/ijerph21081009 (PMC11354845; doi:10.3390/ijerph21081009)
Supplement: Supplementary file 1 [file ijerph-21-01009-s001.zip › ijerph-3076510-supplementary.pdf]

Supplementary Documents for Vision-specific Tools for the Assessment of health-related Quality of Life (HR-QoL) in Children and Adolescents with Visual Impairment: A Scoping Review

ST1: SST Table of resources and how often they appeared on the database searched.

**Included reference**	**Format**	**Database searches (date run: 06/06/2023 and Re-run 26/12/2023)**					**Supplementary resources**
		PubMed	*Science Direct*	*Scopus*	*Web of Science*	*EBSCOhost*	*Other sources were identified through reference review and manual search of Google Scholar.*
**Angeles-Han S, 2011**	Journal			X	X	X	
**Khadka J, 2010**	Journal	X				X	
**Hatt S et al, 2019**	Journal		X				
**Birch E et al, 2004**	Journal	X	X		X	X	
**Gothwal V, 2003**	Journal					X	
**Gothwal V, 2012**	Journal					X	
**Felius J, 2004**	Journal	X	X	X	X		
**Sacchetti M, 2007**	Journal		X		X		
**Tadić V, 2013**	Journal	X	X	X	X	X	
**Cui Y, 2008**	Journal	X		X	X	X	
**Bokhary A, 2013**	Journal	X			X	X	
**Cochrane G, 2011**	Journal	X			X	X	
**Robertson A, 2020**	Thesis	X	X	X	X	X	
**Wadhwani M, 2017**	Journal	X			X	X	
**Cole SR et al, 2001**	Journal		X				
**Carlton J, 2011**	Journal				X		
**Rouse MW et al, 1999**	Journal				X		
**Hatt SR et al, 2010**	Journal				X		
**Andersen CE, 2013**	Thesis						X

**Crescioni M et al, 2014**	Journal			X	X		
**No. Included refs**		9	6	6	14	9	1
**No. Unique refs**		0	1	0	0	2	1
**Total No. refs downloaded**		9	7	6	14	11	1
**No. refs screened**		827	1579	769	829	16557	6
**Sensitivity**		0.45	0.35	0.30	0.70	0.55	0.05
**Precisions**		0.024	0.013	0.026	0.024	0.001	3.333

ST2: List of Domain, Items, and rating scale of the available vision-related tools

Instruments	Source	List of domains/subscales	List of Items	Rating scales
[(VRQOL)-(JIA)-associated uveitis]	Angeles-Han S, 2011	1. Distance<br>2. Near<br>3. Colour<br>4. Night vision<br>5. Functionality<br>6. Photosensitivity<br>7. Optional Driving Domain for children above 16 years<br>8. Global vision<br>9. Physical functioning<br>10. Emotional functioning<br>11. Social functioning<br>12. School functioning	#How often are these tasks hard for you to do?<br>(with glasses or contacts, if you wear them)<br>1. It is hard to see the board if I am sitting in the back of the room.<br>2. It is hard to see the pictures on the television screen from across the room.<br>3. It is hard to see someone's facial expressions (smiling, frowning) when they are talking to me.<br>4. It is hard to see someone's face from across the room.<br>5. It is hard to see the words in my books if I am holding my book far from my face.<br>6. It is hard to see the steps, so I do not trip when going up the stairs.<br>7. It is hard to see how much to fill a glass with a drink.<br>8. It is hard to see where to write on lined notebook paper.<br>9. It is hard adjusting to see where to sit when I walk into a dark room.<br>10. It is hard adjusting to see when I go from a dark room to a brightly lit room.<br>11. It is hard to see when I first walk outside into the sunlight.<br>12. It is hard to play sports that use small balls like baseball, tennis, or golf.<br>13. It is hard to see the cursor on the computer screen.<br>14. It is hard to see words on a tablet or computer if I do not make the font larger.<br>15. It is hard to see text messages on a cell phone.<br>16. It is hard to do activities because of the pain in my eyes.<br>#How often is this true for you?<br>1. I feel left out of activities because of my vision.<br>2. I do not join activities with friends because of my vision.<br>3. I do not like using eye drops.<br>4. I let my eye disease stop me from doing what I want to do.<br>5. I do not like getting injections or infusions for my eye disease.<br>6. I do not like having others know about my eye disease.<br>7. I get frustrated because I cannot do things because of my vision.<br>8. I do not like the way my eyes look.<br>9. I use devices or special aids to help me see, such as:<br>a. large print books, cards, or games?<br>b. Magnifying glass?<br>c. Special lamps or lights?<br>d. Other (not including glasses or contacts):<br>10. I do not like using devices or special aids to help me see (not including glasses or contacts).<br>11. I wear my glasses or contacts like I am supposed to.	6-point scale assessing vision severity.<br>1. excellent<br>2. good<br>3. fair<br>4. poor<br>5. very poor<br>6. Blind.<br>5-point Likert scale assessing task difficulty performing<br>1. Not hard<br>2 A little hard<br>3. Hard,<br>4. Very hard, and<br>5. Cannot do.<br>The child could also choose "does not apply".

Instruments	Source	List of domains/subscales	List of Items	Rating scales
			12. I do not like to wear my glasses or contacts.<br># Global Vision rating:<br>At the present time, my eyesight using both eyes are (with glasses or contacts, if you wear them):	
(CVAQC)	Khadka J, 2010	1. Educational<br>2.near vision<br>3.distance vision<br>4.getting around<br>5.social interaction<br>6. Entertainment<br>7. Sport	Items Because of your eyesight and with your glasses or low vision aids of you use them how difficult do you find…<br>Subscale 1: Education<br>1. Your maths lessons?<br>2. Your science lessons?<br>3. Your geography lessons?<br>4. Your language lessons?<br>Subscale 2: Near vision<br>5. Reading textbooks and work sheets that are given in your school?<br>6. Reading the smallest print in your textbook?<br>7. Drawing, colouring, or painting?<br>8. Reading text messages on your mobile phone?<br>9. Reading restaurant menus?<br>Subscale 3: Distance vision<br>10. Reading the board in your classroom?<br>11. Watching television?<br>12. Watching a film at the cinema?<br>Subscale 4: Getting around<br>13. Going out alone in the daylight?<br>14. Walking in a crowded place?<br>15. Using public transport (bus/train)?<br>16. Reading bus/train timetable on a screen at a station?<br>Subscale 5: Social Interaction<br>17. Chatting with your friends?<br>18. Recognising faces or identifying your friends at arm's length?<br>19. Seeing your friend in a playground?<br>Subscale 6: Entertainment<br>20. Playing video games e.g., a PlayStation?<br>21. Playing computer games?<br>22. Using IPOD/ MP3/MP4 to listen to music? Subscale 7: Sports<br>23. Swimming?<br>24. Taking part in athletics?<br>25. Playing ball games?	6-point Likert scale assessing task difficulty performing<br>1. very easy<br>2. easy<br>3. a little bit difficult<br>4. difficulty<br>5. very difficulty<br>6. Impossible or can't do because of my eyesight.

Instruments	Source	List of domains/subscales	List of Items	Rating scales
(PedEyeQ)	Hatt S et al, 2019	Distance vision,<br>Near vision,<br>Mental distress,<br>Social and Psychological	# PedEyeQ -Functional vision: Child<br>1. Do your eyes make it hard to learn?<br>2. Do you have a hard time seeing?<br>3. Do you have to do things differently than other people because of your eyes?<br>4. Do your eyes make it hard to concentrate?<br>5. Do your eyes make it hard to do certain things?<br>6. Do you have trouble reading close-up?<br>7. Do you have to do certain things to help you see well?<br>8. Is it hard to see the board at school?<br>9. Do you run into things because of your eyes?<br>10. Do your eyes get tired easily?<br># PedEyeQ -Bothered by eyes/vision:<br>1. Does it bother you because your eyes make it hard to learn?<br>2. Does it bother you because your eyes make it hard to play sports?<br>3. Does it bother you because you have a hard time seeing?<br>4. Does it bother you that you can't do certain things because of your eyes?<br>5. Does it bother you because your eyes make it hard to do certain things?<br>6. Does it bother you because it's hard to see the board at school?<br>7. Does it bother you because it's hard to see steps when you walk?<br>8. Does it bother you that bright light makes it hard to do things outside?<br>9. Is taking care of your eye condition hard for you?<br>10. Does it bother you because your eyes hurt?<br># PedEyeQ -Social:<br>1. Does it bother you that it's hard to play/interact with others because of your eyes?<br>2. Does your eye condition cause problems in your family?<br>3. Are you shy because of your eyes?<br>4. Do other people get frustrated with you because of your eyes?<br>5. Do you get teased because of your eyes?<br>6. Do you worry about getting hurt because of your eyes?<br>7. Do you worry about your eyes getting worse?<br>8. Do you worry about what other people think about you because of your eyes?<br>9. Do you worry about getting teased because of your eyes?<br>10. Do you worry about your eyes?<br># PedEyeQ -Frustration / worry:<br>1. Are you bothered by the things you have to do to make your eyes better?<br>2. Do your eyes make you feel unsure of yourself?<br>3. Are you frustrated because your eyes aren't getting better?<br>4. Does it bother you when other people say things or ask questions about your eyes?<br>5. Do you feel "different" because of your eyes?<br>6. Does it bother you that you get extra attention because of your eyes?<br>7. Do you feel left out because of your eyes?	4-point frequency scale.<br>1. Never<br>2. Sometimes<br>3. Most of the time<br>4. All of the time

Instruments	Source	List of domains/subscales	List of Items	Rating scales
			8. Do you worry that your eyes will make it hard to do things when you're older?<br>9. Do you worry about not being able to do things because of your eyes?<br>10. Do you worry about what you might have to do to make your eyes better?	
(CVFQ)	Birch E et al, 2004	General Health<br>General Vision<br>Competence<br>Personality<br>Family Impact<br>Treatment	1. In general, would you say that your child's overall health is:<br>2. at present, would you say that your child's eyesight using both eyes is:<br>3. If your child has an eyesight problem for only one eye, would you say that your child's eyesight in the affected eye is:<br>4. How much of the time do you worry about your child's eyesight?<br>5. How much time do you need to spend on treatment for your child's vision problem (eye doctor appointments, patching, eye drops, and therapy)?<br>6. Does the time you spend on your child's vision problem (eye doctor appointments, patching, eye drops, and therapy) take away from time you would like to spend with your other children or husband/wife?<br>7. Do you and other family members (your spouse or parents) argue about the medical care your child is getting or about the treatment that the doctor has prescribed?<br>8. I am afraid that my child will never have good vision.<br>9. I am bothered by other people's comments about my child's vision or eyes when I take him/her to a store or mall.<br>10. My child likes to try new things.<br>11. Taking my child to the eye doctor is stressful.<br>12. I think that my child's vision will improve.<br>13. My child feels different from other children.<br>14. My child is happy most of the time.<br>15. I notice other children looking at my child.<br>16. My child likes to visit with relatives.<br>17. My child is teased because of his/her vision problems.<br>18. My child cries a lot.<br>19. I worry that my child may not be able to read, watch TV, or drive a car.<br>20. My child makes new friends easily.<br>21. My child is affectionate.<br>22. My child gets along well with our other children and friends.<br>23. My child gets angry or frustrated because of his vision problem.<br>24. We stay at home a lot because of my child's vision problem.<br>25. My child can feed himself/herself.<br>26. My child plays with toys.<br>27. My child can recognize faces (friends, relatives) across a room.<br>28. My child can imitate others (make a face, stick tongue out, play peek-a-book).<br>29. My child can dress himself/herself.<br>30. My child can brush his/her teeth.<br>31. My child can wash his/her face.<br>32. My child adjusts to changes in lighting (going out into bright sunlight or entering a	5-points scales<br>Rating visual status.<br>1. excellent<br>2. very good<br>3. good<br>4. fair<br>5. poor),<br><br>5-point agreement scale.<br>1. strongly disagree<br>2. disagree<br>3. agree<br>4. strongly agree<br>5. does not apply to my child)<br><br>5-point scale assessing task difficulty<br>1. No difficulty<br>4. difficult<br>5. cannot do this because of eyesight)<br><br>5-point frequent scale<br>1. never<br>2. always<br>3. my child too young to attempt this

Instruments	Source	List of domains/subscales	List of Items	Rating scales
			dark room or theatre.)<br>33. My child can ride a bicycle.<br>34. My child can play a sport or active game (for example, tag).<br>35. My child will track a mobile or a moving toy.<br>36. My child can locate a small piece of food (a raisin or Cheerio) and grasp it.<br>37. My child can pour liquid into a cup or glass.<br>38. My child can dial a telephone.<br>39. My child helps with chores.<br>40. My child can tell what time it is.<br>41. My child can identify coins.<br>42. My child enjoys looking at books.<br>43. My child is interested in playing with our pet(s).<br>44. My child has a regular sleep routine.<br>45. My child's eyesight makes it difficult for him/her to learn to walk, run, skip, or jump.<br>46. My child's vision gets in the way of his/her learning.<br>47. My child's eyesight has made it difficult for him/her to learn to read.<br>48. My child enjoys watching television, videos, or playing video games.<br>49. My child likes to travel on family vacations.<br>50. My child enjoys playing with others (sisters and brothers or friends).<br>51. My child enjoys drawing, painting, or other art activities.<br>52. My child's eyesight makes it difficult for him/her to find something on a crowded shelf or in a closet.<br>53. My child makes eye contact with me and smiles.<br>54. My child bumps into people, walls, or furniture.<br>55. My child trips over curbs or steps.<br>56. My child bumps into other people.<br>57. I have trouble applying treatment (for example, putting on an eye patch or glasses, giving eye drops or other medication).<br>58. My child is uncomfortable when treated (for example, while wearing a patch or glasses or when you put in eye drops).<br>59. My child is less active when treated (for example, when wearing a patch or glasses, or when taking eye drops or medication).<br>60. I worry when my child refuses treatment (for example, pulls off the patch or glasses, or squeezes eye shut when trying to put in eye drops).<br>61. I sometimes forget to treat my child.	
(LVP-FVQ)	Gothwal V, 2003	Distance vision,<br>Near vision,<br>Colour vision	1. Do you have any difficulty in making out whether the person you are seeing across the read is a boy or a girl, during the day?<br>2. Do you have any difficulty in seeing whether somebody is calling you by waving his or	5-point scale

Instruments	Source	List of domains/subscales	List of Items	Rating scales
		and<br>Visual field	her hand from across the road?<br>3. Do you have difficulty in walking alone in the corridor at school without bumping into objects or people?<br>4. Do you have any difficulty in walking home at night (from tuition or a friend's house) without assistance when there are streetlights?<br>5. Do you have any difficulty in copying from the black- board while sitting on the first bench in your class?<br>6. Do you have difficulty in reading the bus numbers?<br>7. Do you have any difficulty in reading the other details on the bus (such as its destination?)<br>8. Do you have any difficulty in reading your textbooks at an arm's length?<br>9. Do you have any difficulty in writing along a straight line?<br>10. Do you have any difficulty in finding the next line while reading when you take a break and then resume reading?<br>11. Do you have any difficulty in locating dropped objects (pen, pencil, and eraser) within the classroom?<br>12. Do you have any difficulty in threading a needle?<br>13. How much difficulty do you have in distinguishing be- between 1-rupee and 2-rupee coins (without touching)?<br>14. Do you have difficulty in climbing up or down stairs?<br>15. Do you have difficulty in lacing your shoes?<br>16. Do have difficulty locating a ball while playing in the daylight?<br>17. Do you have difficulty applying paste on your toothbrush?<br>18. Do you have difficulty in locating food on your plate while eating?<br>19. Do you have difficulty in identifying colours (e.g., while colouring)?<br>20. How do you think your vision is compared with that of your normal-sighted friend? Do you think your vision is?	(0-no difficulty, 4-cannot do the task due to my eyesight)<br>Yes/ No option was chosen first, if the respondent chose No option, their score was zero, which means there were no difficulties. However, if Yes was chosen, then they would have to rate the level of difficulties from:<br>1. little difficult<br>2. difficult<br>3. more difficult<br>4. unable to do the activity due to visual reasons.
(LVP-FVQ II)	Gothwal V, 2012	Activities of daily living,<br>Academic and leisure activities	# Do you have difficulty in....?<br>1. Walking without bumping into objects/people<br>2. Getting around alone in places you know at night-time<br>3. Reading the bus numbers<br>4. Finding out the next line while reading<br>5. Locating dropped objects<br>6. Threading a needle<br>7. Locating ball while playing in the daytime<br>8. Copying from your friend's notebook<br>9. Reading the rates on items (e.g., chips packets, biscuit packets)<br>10. Reading the phone numbers on mobile phone<br>11. Watching TV	Three-category scale<br>1. No difficulty.<br>2. some difficulty and<br>3. a lot of difficulty)<br>The response options of the global rating item were as follows:<br>1. as well as your friend's<br>2. a little bit worse than your friend's

Instruments	Source	List of domains/subscales	List of Items	Rating scales
			12. Seeing time in your wristwatch<br>13. Moving around safely in places you don't know in the daytime<br>14. Moving around safely in places you don't know at night-time<br>15. Reading from a computer screen<br>16. Copying the small letters from the board<br>17. Walking on uneven ground<br>18. Reading shop names<br>19. Seeing animals in the zoo<br>20. Identifying dirt, stains on your clothes<br>21. Reading your books at near<br>22. Seeing your friend in the playground while playing<br>23. Watching a movie at the theatre<br>24. Selecting a song using an iPod<br>25. Playing video games<br>26. Seeing the numbers and markings on the scale<br># How do you think about......?<br>27. Your vision compared to your normally sighted friend	3. Much worse than your friend.
[(VRQOL) In young children]	Felius J, 2004	General Health<br>General Vision<br>Competence<br>Personality<br>Family Impact<br>Treatment	1. In general, would you say that your child's overall health is: [Q]<br>2. at present, would you say that your child's eyesight using both eyes is: [Q]<br>3. If your child has an eyesight problem for only one eye, would you say that your child's eyesight in the affected eye is: [Q] [NA]<br>4. How much of the time do you worry about your child's eyesight? [F]<br>5. How much time do you need to spend on treatment for your child's vision problem (eye doctor appointments, patching, eye drops, and therapy)? [F]<br>6. Does the time you spend on your child's vision problem (eye doctor appointments, patching, eye drops, and therapy) take away from time you would like to spend with your other children or husband/wife? [F]<br>7. Do you and other family members (your spouse or parents) argue about the medical care your child is getting or about the treatment that the doctor has prescribed? [F]<br>8. I am afraid that my child will never have good vision. [A] [NA]<br>9. I am bothered by other people's comments about my child's vision or eyes when I take him/her to a store or mall. [A] [NA]<br>10. My child likes to try new things. [A] [NA]<br>11. Taking my child to the eye doctor is stressful. [A] [NA]<br>12. I think that my child's vision will improve. [A] [NA]<br>13. My child feels different from other children. [A] [NA]<br>14. My child is happy most of the time. [A] [NA]<br>15. I notice other children looking at my child. [A] [NA]<br>16. My child likes to visit with relatives. [A] [NA]<br>17. My child is teased because of his/her vision problems. [A] [NA]<br>18. My child cries a lot. [A] [NA]	5-point scale<br>QoL scale:<br>1. excellent,<br>2. very good<br>3. good,<br>4. fair,<br>5. bad,<br>6. very bad<br>Frequency scales: 1. never,<br>2. once in a while<br>3. sometimes<br>4. Often<br>5. always<br>Agreement scales: 1. strongly agree<br>2. agree,<br>3. neutral,<br>4. disagree.<br>5. strongly disagree<br>Difficulty:<br>1. difficult,<br>2. little difficulty<br>3. easy

Instruments	Source	List of domains/subscales	List of Items	Rating scales
			19. I worry that my child may not be able to read, watch TV, or drive a car. [A] [NA]<br>20. My child makes new friends easily. [A] [NA]<br>21. My child is affectionate. [A] [NA]<br>22. My child gets along well with our other children and friends. [A] [NA]<br>23. My child gets angry or frustrated because of his vision problem. [A] [NA]<br>24. We stay at home a lot because of my child's vision problem. [A] [NA]<br>25. My child can feed himself/herself. [D] [TY]<br>26. My child plays with toys. [D] [TY]<br>27. My child can recognize faces (friends, relatives) across a room. [D] [TY]<br>28. My child can imitate others (make a face, stick tongue out, play peek-a-boo). [D] [TY]<br>29. My child can dress himself/herself. [D] [TY]<br>30. My child can brush his/her teeth. [D] [TY]<br>31. My child can wash his/her face. [D] [TY]<br>32. My child adjusts to changes in lighting (going out into bright sunlight or entering a dark room or theatre.) [D] [TY]<br>33. My child can ride a bicycle. [D] [TY]<br>34. My child can play a sport or active game (for example, tag). [D] [TY]<br>35. My child will track a mobile or a moving toy. [D] [TY]<br>36. My child can locate a small piece of food (a raisin or Cheerio) and grasp it. [D] [TY]<br>37. My child can pour liquid into a cup or glass. [D] [TY]<br>38. My child can dial a telephone. [D] [TY]<br>39. My child helps with chores. [D] [TY]<br>40. My child can tell what time it is. [D] [TY]<br>41. My child can identify coins. [D] [TY]<br>42. My child enjoys looking at books. [A] [TY]<br>43. My child is interested in playing with our pet(s). [A] [TY]<br>44. My child has a regular sleep routine. [A] [TY]<br>45. My child's eyesight makes it difficult for him/her to learn to walk, run, skip, or jump. [A] [TY]<br>46. My child's vision gets in the way of his/her learning. [A] [TY]<br>47. My child's eyesight has made it difficult for him/her to learn to read. [A] [TY]<br>48. My child enjoys watching television, videos, or playing video games. [A] [TY]<br>49. My child likes to travel on family vacations. [A] [TY]<br>50. My child enjoys playing with others (sisters and brothers or friends). [A] [TY]<br>51. My child enjoys drawing, painting or other art activities. [A] [TY]<br>52. My child's eyesight makes it difficult for him/her to find something on a crowded shelf or in a closet. [A] [TY]<br>53. My child makes eye contact with me and smiles. [F] [TY]<br>54. My child bumps into people, walls, or furniture. [F][TY]<br>55. My child trips over curbs or steps. [F] [TY]<br>56. My child bumps into other people. [F] [TY]	4. very easy<br>5. Cannot perform the task due to eyesight.

Instruments	Source	List of domains/subscales	List of Items	Rating scales
			57. I have trouble applying treatment (for example, putting on an eye patch or glasses, giving eye drops or other medication). [F]<br>58. My child is uncomfortable when treated (for example, while wearing a patch or glasses or when you put in eye drops). [F]<br>59. My child is less active when treated (for example, when wearing a patch or glasses, or when taking eye drops or medication). [F]<br>60. I worry when my child refuses treatment (for example, pulls off the patch or glasses, or squeezes eye shut when trying to put in eye drops). [F]<br>61. I sometimes forget to treat my child. [F]	
(QUICK)	Sacchetti M, 2007	Physical well-being<br>Emotional well-being<br>Self-esteem<br>Family<br>Friends<br>School<br>Disease	#During the Last Two Weeks, Because of Conjunctivitis...<br>1. You felt burning in your eyes?<br>2. You had trouble staying in air-conditioned rooms?<br>3. You had to use tissues<br>4. You had puffy eyes<br>5. You had problems in the light<br>6. You had tearing<br>7. You had itchy eyes<br>8. You had red eyes<br>9. You had blurred vision<br>10. You had eye secretion<br>11. You had to use eye drops<br>12. You had, in the morning, closed and sticky eyes<br>13. You had trouble playing outdoors<br>14. You had trouble practicing sports (football, gym)<br>15. You had trouble meeting your friends<br>16. You had trouble going to the swimming pool	3-point frequency scale:<br>1. Never,<br>2. Sometimes, and<br>3. Always
(FVQ-CYP)	Tadić V, 2013	Functioning, home, school and leisure	1. Watching TV<br>2. Playing video and computer games<br>3. Playing other games, e.g., board games or card games<br>4. Using the computer for homework<br>5. Reading food packets, labels, or recipes<br>6. Doing household chores, e.g., washing up<br>7. Telling the time on a wristwatch<br>8. Telling the time on a wall clock<br>9. Using the computer for lessons<br>10. Reading small print textbooks, worksheets, and exam papers<br>11. Reading enlarged textbooks, worksheets, and exam papers<br>12. Drawing or painting<br>13. Reading handwriting<br>14. Seeing the board in the class<br>15. Recognizing people, e.g., in school corridors	5 response options<br>1. Very easy;<br>2. Easy.<br>3. Difficult.<br>4. Very difficult or impossible<br>5. this doesn’t apply to me/I don’t do this for other reasons)

Instruments	Source	List of domains/subscales	List of Items	Rating scales
			16. Recognizing other people's facial expressions<br>17. Finding friends in the playground<br>18. Taking part in science classes<br>19. Taking part in geography classes<br>20. Taking part in math classes<br>21. Taking part in Physical Education<br>22. Taking part in English classes<br>23. Keeping up with the teacher in lessons<br>24. Keeping up with other students in class<br>25. Getting around the school by yourself<br>26. Getting around outdoors by yourself<br>27. Reading signs and posters at stations or shops<br>28. Getting around in crowds by yourself<br>29. Seeing small moving objects, e.g., balls<br>30. Seeing large moving objects, e.g., cars passing Using<br>31. Using the escalators<br>31. Playing team sports, e.g., football, without adaptations<br>32. Watching films in the cinema<br>33. Watching plays and shows in the theatre<br>34. Reading price tags<br>35. Finding the correct money to pay	
[(VRQOL) in children with ID]	Cui Y, 2008	1. School activities<br>2. Health<br>3. Social life<br>4. Leisure activities<br>5. Family life<br>6. Academic performance<br>7. Gross motor activities<br>8. Fine motor activities<br>9. Object discrimination<br>10. Distance vision<br>11. Treatment<br>12. General health<br>13. General vision<br>14. Competence<br>15. Personality<br>16. Family Impact	1. When I am having dinner with my family, I feel<br>2. When I go to bed at night, I feel<br>3. My brothers and sisters make me feel<br>4. When I am with my grandparents, I feel<br>5. When I watch television, I feel<br>6. When I am eating, I feel<br>7. When I go to the doctor's, I feel<br>8. When I stay in hospital, I feel<br>9. When I take medicine, I feel<br>10. When I am sick, I feel<br>11. When I am not sick, I feel<br>12. On my birthday, I feel<br>13. When I play alone, I feel<br>14. When it is a holiday, I feel<br>15. When people tell me what to do, I feel<br>16. At school, I feel<br>17. When I make a drawing, I feel<br>18. When I move (walk, run, jump), I feel<br>19. When I am playing outside, I feel<br>20. When I play a sport, I feel	4-point Likert scale<br>1. Not happy at all<br>2.Not happy<br>3.Happy<br>4. Very Happy<br>And yes or No

Instruments	Source	List of domains/subscales	List of Items	Rating scales
			21. When I do my homework, I feel<br>22. When I read a book, I feel<br>23. When my work is marked at school, I feel<br>24. Can you climb up and down stairs?<br>25. Do you bump into things?<br>26. Can you kick a ball when you play?<br>27. Can you hit the ball when you play?<br>28. Can you find food on your plate when eating?<br>29. Can you find your favorite toy at playtime?<br>30. Can you pick up a red pencil from a box of pencils?<br>31. Can you put toothpaste on your toothbrush by yourself?<br>32. Can you do (tie) up your shoelaces by yourself?<br>33. Can you see the TV clearly?<br>34. Can you see a person's face across the road?<br>35. Can you see the bus numbers clearly?<br>36. Can you see the pictures in your books clearly?<br>37. Can you read a book by yourself?<br>38. Can you draw a straight line on paper without a ruler?<br>39. Can you copy from the board in class?<br>40. Can you write the numbers from 1 to 10?<br>41. Can you write your name?<br>42. Can you remember words easily?<br>43. Can you finish your homework on time?<br>44. Can you do math?<br>45. Is your handwriting neat?<br>46. In general, would you say that your child's overall health is:<br>47. At present, would you say that your child's eyesight (using both eyes) is:<br>48. If your child has an eyesight problem for only one eye, would you say that your child's eyesight in the affected eye is:<br>49. My child can feed himself/herself.<br>50. My child can recognize faces (friends, relatives) across a room.<br>51. My child can dress himself/herself.<br>52. My child can brush his/her teeth.<br>53. My child can wash his/her face.<br>54. My child can ride a bicycle.<br>55. My child can play a sport or an active game (for example, football).<br>56. My child can locate a small piece of food (a raisin or Cheerio) and grasp it.	

Instruments	Source	List of domains/subscales	List of Items	Rating scales
			57. My child can pour liquid into a cup or glass.<br>58. My child can dial a telephone.<br>59. My child helps with chores.<br>60. My child can tell what time it is.<br>61. My child can identify coins.<br>62. My child's eyesight makes it difficult for him/her to find something on a crowded shelf or in a closet.<br>63. My child bumps into people, walls, or furniture.<br>64. My child trips over curbs or steps.<br>65. My child is happy most of the time.<br>66. My child likes to visit with relatives.<br>67. My child makes friends easily.<br>68. My child is affectionate.<br>69. My child gets along well with our other children and friends.<br>70. My child enjoys looking at books.<br>71. My child's eyesight makes it difficult for him/her to learn to walk, run, skip, or jump.<br>72. My child's vision gets in the way of his/her learning.<br>73. My child enjoys watching television, videos, or playing video games.<br>74. My child likes to travel on family vacations.<br>75. My child enjoys playing with others (sisters and brothers or friends).<br>76. My child enjoys drawing, painting or other art activities.<br>77. How much of the time do you worry about your child's eyesight?<br>78. How much time do you need to spend on treatment for your child's vision problem (eye doctor appointment, patching, eye drops and therapy)?<br>79. Does the time you spend on your child's vision problem (eye doctor appointments, patching, eye drops, and therapy) take away from time you would like to spend with your other children or husband/wife?<br>80. Do you and other family members (your spouse or parents) argue about the medical care your child is getting or about treatment that the doctor has prescribed?<br>81. I am bothered by other people's comments about my child's vision or eyes when I take him/her to a store or mall.<br>82. My child feels different from other children.<br>83. I notice other children looking at my child.<br>84. My child is teased because of his/her vision problem.<br>85. I worry that my child may not be able to read, watch TV, or drive	

Instruments	Source	List of domains/subscales	List of Items	Rating scales
			a car.<br>86. I have trouble applying eye/vision treatment (for example, putting on an eye patch or glasses, giving eye drops or other medication).<br>87. My child is uncomfortable when treated (for example, while wearing a patch or glasses or when you put in eye drops).<br>88. My child is less active when having treatment (for example, when wearing a patch or glasses, or when taking eye drops or medication).<br>89. I worry when my child refuses treatment (for example, pulls off the patch or glasses, or squeezes eye shut when trying to put in eye drops).<br>90. I forget to give my child treatment.	
(CVLS)	Bokhary A, 2013	mood,<br>self-esteem,<br>social relations,<br>functional vision,<br>Visio-motor and school performance	1. Usually, how happy do you feel?<br>2. How much do you think that you are good looking?<br>3. How much do you feel that other children want to be your friend?<br>4. If you have a sister or brother, how much do you enjoy playing with them?<br>5. Do you have many friends?<br>6. How easy is it for you to make new friends?<br>7. How clearly (well) can you see the picture on your TV?<br>8. How clearly (well) can you see the moving picture in hand-held computer games?<br>9. How clearly (well) can you see pictures in a book?<br>10. How easy is it for you to put the pen cap back on?<br>11. How easy is it for you to cut shapes with scissors?<br>12. How easy is it for you to draw a straight line with a ruler?<br>13. How good are you at writing exactly on the line?<br>14. How easy is it for you to see steps when you walk up or down stairs?<br>15. How often do you do the right thing?<br>16. How much do you enjoy reading the smallest print in your textbook?<br>17. How easy is it for you to draw, colour in a picture or write words at school?<br>18. How clearly (well) can you see the smallest writing on the board at school?<br>19. When you read a story, how easy is it for you to explain it to someone else?<br>20. How clever do you think you are?<br>21. How easy is it for you to answer the questions in the class at school?	5-point Likert scale with five categories<br>1.Not at all<br>2. A little<br>3. Moderately<br>4. A lot<br>5. Extremely
(IVI_C)	Cochrane G, 2011	NR	7. Are you confident that you can move around safely in places you don't know at night-time?<br>5. Are you confident in places you don't know?<br>16. Do you get frustrated?<br>26. When you ask for help, do people understand how much help you need?<br>4. Are you confident to use public transport (such as buses, trains, ferries) by yourself?<br>8. Can you find your friends in the playground at lunch and play time?<br>18. Do other students understand your special needs?	5-point frequency score response<br>5. always<br>4. almost always<br>3. some- times<br>2. almost never<br>1. never

Instruments	Source	List of domains/subscales	List of Items	Rating scales
			27. Do people tell you that you can't do the things that you want to do?<br>6. Are you confident that you can move around safely in places you don't know in the daytime?<br>9. When you are in a room, can you recognise people you know before they speak to you?<br>12. Has your eyesight stopped you from doing the things that you want to do?<br>14. Do other students help you to join in with them?<br>15. Do you find it hard to join in with other students?<br>11. Do you get the chance to go to activities other than sport (such as social groups)?<br>21. Do you get all the information at the same time as the other students?<br>22. Do you get enough time in school to complete the work set by the teacher?<br>10. Can you take part in games or sports that you want to play with your friends?<br>3. Are you confident to make your own way to school?<br>13. Do other students help you when you ask them for help?<br>23. When you are in the classroom, are you confident about asking for help you need?<br>28. Do people stop you from doing the things you want to do?<br>2. Do you find it difficult to go downstairs or to step off the footpath?<br>20. In the classroom, do you get all the same information as other students?<br>19. Do your teachers understand your special needs?	
[(PRO-FV) for children and young people with VI]	Robertson A, 2020	home,<br>school and leisure,<br>restrictions and limitations,<br>levels of functioning,<br>mobility, and communication	1. Watching TV<br>2. Playing video and computer games<br>3. Playing indoor games, such as board games or card games<br>4. Using the computer at home to do my homework<br>5. Reading food packets, tickets, labels or recipes<br>6. Doing household chores, for example, washing up or tidying my bedroom<br>7. Looking after my appearance, for example, doing my hair, shaving, or putting on make-up<br>8. Making myself a snack at home<br>9. Making myself a meal<br>10. Finding objects, I have dropped such as coins or glasses on a low-contrast surface<br>11. Using the computer at school or college to do schoolwork/ coursework<br>12. Reading small print textbooks, worksheets, and exam papers<br>13. Reading other people's handwriting<br>14. Seeing the board in the classroom when sitting at the front<br>15. Recognising people, for example, in corridors at school/college or shops<br>16. Recognising other people's facial expressions when they are close to me/at arm's length<br>17. Finding friends in crowded areas<br>18. Doing maths<br>19. Doing science<br>20. Doing sports at school/college<br>21. Keeping up with the teacher or tutor in lessons	4-point scale to assess task difficulties.<br>1. Very easy<br>2. Easy<br>3: A bit difficult<br>4. Very difficult or impossible

Instruments	Source	List of domains/subscales	List of Items	Rating scales
			22. Keeping up with other students in lessons<br>23. Getting around school/college by myself<br>24. Playing team sports, such as football, without adaptations such as special balls<br>25. Seeing small balls when playing games, such as tennis or cricket<br>26. Seeing big moving objects, such as bikes passing, in daylight<br>27. Getting around outdoors e.g., shops or the park, by myself when it's daylight<br>28. Getting around outdoors e.g., shops or the park, by myself when it's dark<br>29. Getting around in crowds by myself<br>30. Finding my way around an unfamiliar house or a new building<br>31. Reading signs and posters at stations or shops<br>32. Finding correct money to pay when shopping<br>33. Watching films in the cinema<br>34. Watching shows, such as plays at the theatre<br>35. Crossing the road by myself<br>36. Using public transport, such as trains, buses, or the tube by myself<br>37. Using a mobile phone to text people<br>38. Using a mobile phone or tablet for social networking, for example, Facebook, Twitter or Myspace<br>39. Reading enlarged worksheets and textbooks like dictionaries<br>40. Drawing or painting<br>41. Playing outdoor games, such as tag or hide and seek.	
(CHVI-VFQ)	Wadhwani M, 2017	mobility,<br>education,<br>daily routine<br>Psychosocial	*Final questionnaire in the age group of 5 to 9 years:<br>1. Does your child has any difficulty in walking alone in the corridor at school without bumping into objects or people<br>2. Does your child has any difficulty in copying from blackboard while sitting on front bench in your class?<br>3. Does your child has any difficulty in reading the bus number?<br>4. Does your child has any difficulty in reading your textbooks at arm's length?<br>5. Does your child has any difficulty in writing along a straight line?<br>6. Does your child has any difficulty in finding the next line while reading when you take a break and then resume reading?<br>7. Does your child has any in locating the dropped objects within the class room?<br>8. Does your child has any difficulty in locating/identifying food on your plate while eating?<br>9. Because of vision how much problem does your child have in seeing if there are animals or vehicles when walking?<br>10. Because of vision how much problem does your child have in going out at night?<br>11. Because of vision how much problem does your child has in locking or unlocking the door?	5-point Likert scale assessing the severity of the disability. (Range 0–4), where a score of 4 represented severe disability.<br>4-point scale assessing the level of difficulty. 0-3 were higher score means greater difficulty.

Instruments	Source	List of domains/subscales	List of Items	Rating scales
			12. Because of vision how much problem does your child has in seeing differences in colours?<br>13. Because of vision how much problem does your child has in going to social functions such as weddings/birthday parties?<br>14. Because of vision how much problem does your child has in finding your way indoors?<br>15. Because of eye problem do you feel frightened that your child may lose remaining vision?<br>16. Does your child has reduced vision?<br>*In the age group of 10 to 15 years:<br>1. Because of your vision how much problem do you have in finding your way indoors?<br>2. Because of your vision how much problem do you have in seeing differences in colours?<br>3. Do you have difficulty in applying tooth paste on your toothbrush?<br>4. Because of your vision how much problem do you have in making out differences in coins or notes?<br>5. Do you have any difficulty in climbing up and down the stairs?<br>6. Because of your vision how much problem do you have in going to the toilet?<br>7. Do you have any difficulty in locating/identifying food on your plate while eating?<br>8. Do you have difficulty in lacing your shoe?<br>9. Because of your vision how much problem do you have in seeing the steps of the bus when climbing in or out?<br>10. Because of your vision how much problem do you have in seeing if there are animals or vehicles when walking?<br>11. Because of your vision how much problem do you have in going to social functions such as weddings/birthday parties?<br>12. Because of your vision how much problem do you have in going out at night?<br>13. Do you have difficulty in locating a ball while playing with it in day light?<br>14. Because of your vision how much problem do you have in recognising the face of a person standing near you?<br>15. Do you have any difficulty in finding the next line while reading when you take a break and then resume reading?<br>16. Do you have any difficulty in writing along a straight line?<br>17. Do you have any difficulty in reading your textbooks at arm's length?<br>18. Because of your vision how much problem do you have in seeing when coming into the house after being in the sunlight?<br>19. Because of your eye problem do you ashamed that you can't see?<br>20. Because of your vision how much problem do you have in seeing outside in bright sunlight?<br>21. Because of your vision how much problem do you have in recognising people from a distance?<br>22. Do you have any difficulty in reading the bus number?	

Instruments	Source	List of domains/subscales	List of Items	Rating scales
			23. Do you have any difficulty in copying from blackboard while sitting on front bench in your class?<br>24. How do you think your vision is compared with that of your normal-sighted friend?<br>25. Do you have reduced vision?<br>26. Because of your eye problem do you feel frightened that you may lose your remaining vision?<br>27. Because of your eye problem do you feel you have become a burden on others?	
(ATI)	Cole SR et al, 2001	Parent version: domain relating to treatment of amblyopia:<br>1. Adverse effects,<br>2. Treatment compliance and<br>3. Social stigma<br>Child version: had domains relating to the treatment of amblyopia:<br>1. Adverse effects,<br>2. Treatment compliance<br>The Amblyopia Treatment Index (ATI) added a domain number.<br>3. Functioning at near	# Patching questionnaire<br>1. My child does not seem to mind wearing the patch once it is on.<br>2. I worry that by wearing the patch, my child may miss out on fun activities (such as games and parties).<br>3. wearing patch the patch affects my child's learning<br>4. Wearing the patch makes it hard for my child to play outside, such as running, jumping, or riding a bike or tricycle.<br>5. I have trouble putting on my child's patch and keeping it on.<br>6. wearing a patch is a source of tension or conflict in my relationship:<br>A. with my child.<br>B. with my child's babysitter or teacher.<br>7. Wearing the patch makes it difficult for my child to draw, colour, or write.<br>8. I worry that my child will become injured when wearing the patch.<br>9. My child can see well when wearing the patch.<br>10. My child complains when it is time to wear the patch.<br>11. Wearing the patch makes my child's eyes or eyelids red or irritated.<br>12. I worry that my child does not wear the patch enough.<br>13. My child is clumsier and more uncoordinated than usual when wearing the patch.<br>14. I notice that other children stare at my child when the patch is on.<br>15. I believe that wearing a patch will improve my child's vision.<br>16. Wearing the patch makes it difficult for my child to play with blocks or toys.<br>17. I sometimes forget to put the patch on my child.<br>18. I worry that wearing the patch will make my child feel different from other children.<br># Atropine questionnaire<br>1. My child does not seem to mind using the drops.<br>2. I worry that by using drops, my child may miss out on fun activities (such as games and parties).<br>3. Using the drops affects my child's learning<br>4. Using the drops makes it hard for my child to play outside such as running, jumping, or riding a bike or tricycle.<br>5. I have trouble putting the drops in my child's eye.<br>6. Using the drops is a source of tension or conflict in my relationship:<br>A. with my child.	5-point agreement scales:<br>1. strongly agree<br>2. agree<br>3. neutral<br>4. Strongly disagree 5. Does not apply.

Instruments	Source	List of domains/subscales	List of Items	Rating scales
			B. with my child's babysitter or teacher.<br>7. Using the drops makes it difficult for my child to draw, colour, or write.<br>8. I worry that my child will become injured when using the drops.<br>9. My child can see well when using drops.<br>10. My child complains when it is time to put in the drops.<br>11. Using the drops makes my child's eyes or eyelids red or irritated.<br>12. I worry that my child does not get the drops often enough.<br>13. My child is clumsier and more uncoordinated than usual when using the drops.<br>14. I notice that other children stare at my child when the drops are in.<br>15. I believe that using the drops will improve my child's vision.<br>16. Using the drops makes it difficult for my child to play with blocks or toys.<br>17. I sometimes forget to put the drops in my child.<br>18. I worry that using the drops will make my child feel different from other children.	
(CAT-QoL)	Carlton J, 2011/2013		1. Are you feeling sad due to the patch?<br>2. feeling of your patch on your face (like sticky, or itchy)<br>3. Are you hurt by the results of the patch?<br>4. does the patch affect you when you're doing work at school (like reading and writing)<br>5. Cross<br>6. How other children have treated you (like laughing at you, or calling you names) because of your patch<br>7. does the patch affect you when doing things (like playing on the computer, colouring, playing games, watching TV)<br>8. are you worried about the results of the patch<br>9. Are you upset about the results of the patch?<br>10. Does the patch affect your play with my friends?<br>11. Does the patch affect your happiness?	5-point scale:<br>1. Not,<br>2. Little bit,<br>3. A bit,<br>4. Quite a bit,<br>5. A lot/ really.
(CISS)	Rouse MW et al, 1999	The unidimensional scale measuring symptom severity	1. Do your eyes feel tired when reading or doing close work?<br>2. Do your eyes feel uncomfortable when reading or doing close work?<br>3. Do you have headaches when reading or doing close work?<br>4. Do you feel sleepy when reading or doing close work?<br>5. Do you lose concentration when reading or doing close work?<br>6. Do you have trouble remembering what you have read?<br>7. Do you have double vision when reading or doing close work?<br>8. Do you see the words move, jump, swim, or appear to float on the page when reading or doing close work?<br>9. Do you feel like you read slowly?	5-point frequency scale.<br>0. Never<br>1. Infrequently/not very often<br>2. Sometimes<br>3. Fairly often<br>4. Always

Instruments	Source	List of domains/subscales	List of Items	Rating scales
			10. Do your eyes ever hurt when reading or doing close work?<br>11. Do your eyes ever feel sore when reading or doing close work?<br>12. Do you feel a "pulling" feeling around your eyes when reading or doing close work?<br>13. Do you notice the words blurring or coming in and out of focus when reading or doing close work?<br>14. Do you lose your place while reading or doing close work?<br>15. Do you have to re-read the same line of words when reading?	
(IXTQ)	Hatt SR et al, 2010	Functional<br>Psychosocial<br>Surgery	# children and proxy<br>1. I worry about my eyes<br>2. It bothers me that people wonder what is wrong with my eyes<br>3. It bothers me because I have to wait for my eyes to clear up<br>4. Kids tease me because of my eyes<br>5. I am bothered when grownups say things about my eyes<br>6. I am bothered when my parents say things about my eyes<br>7. It bothers me that I have to shut one eye when it is sunny<br>8. I feel different from other kids because my eyes go in and out<br>9. I worry about what other people think of me because of my eyes<br>10. My eyes make it hard to look people in the eye<br>11. It is hard to concentrate because of my eyes<br>12. My eyes make it hard for me to make friends<br># Parents version<br>1. I worry about my child's eyes<br>2. I worry that my child will be less independent because of his/her eyes<br>3. I worry that my child will have permanent damage to his/her eyes<br>4. I worry that my child doesn't see well<br>5. I worry about how my child's eyes will affect him/her socially<br>6. I worry that my child will get hurt physically because of his/her eyes<br>7. I worry about the possibility of surgery 8. I worry about my child becoming self-conscious because of his/her eyes.<br>9. I worry that my child will not be able to see the board at school<br>10. I worry about other kids teasing my child because of his/her eyes<br>11. It worries me what others will think about my child because of his/her eyes 12. I worry that my child's eye condition will affect his/her personality.<br>13. I worry that my child's eyes will affect his/her social life if nothing is done<br>14. I worry about my child's eyesight long term<br>15. I worry about my child's depth perception<br>16. I worry about whether or not my child should have surgery<br>17. I worry about my child's ability to make friends.	3-point frequency scale:<br>100. A lot or almost<br>50. always and<br>0. Not at all/ never<br>5-point frequency scale for older children and parents<br>100. Never<br>75. Almost never<br>50. Sometimes<br>25. Often<br>0. Almost Always
(PREP2)	Andersen CE, 2013	1.overall vision<br>2. Near vision	1. My vision is very clear when I look far away (movies or board at school).<br>2. My eyes are sometimes uncomfortable.	5-point agreement scale.

Instruments	Source	List of domains/subscales	List of Items	Rating scales
		3. far vision<br>4. symptoms<br>5. appearance<br>6. satisfaction<br>7. activities<br>8. academics<br>9. handling<br>10. peer perception	3. I am happy with the way that I look.<br>4. When I play sports or other activities, I sometimes don't wear vision correction because it bothers me.<br>5. When I play outdoors, I never have a problem with my vision correction.<br>6. My friends make fun of me because of my vision correction.<br>7. I love my vision correction.<br>8. When I look far away, my vision is not as clear as I would like it to be.<br>9. My eyes are always comfortable.<br>10. I do not like how I look when I wear my vision correction.<br>10. I do not like how I look when I wear my vision correction.<br>11. When I play outdoors, I never have a problem with my vision correction.<br>12. My vision correction sometimes breaks or falls off while I am wearing it.<br>13. My friends want the same kind of vision correction that I have.<br>14. I don't like my vision correction very<br>Much<br>15. My vision is very clear when I look at something close (books or cell phones).<br>16. My eyes sometimes itch, burn, or feel.<br>17. My vision correction makes me look cool.<br>18. When I play outside, my vision correction sometimes bothers me.<br>19. When I am active, my vision correction never falls off.<br>20. My friends don't like how I look when I wear my vision correction.<br>21. I never have problems with my vision correction.<br>22. When I read, my vision is not as clear as I would like it to be.<br>23. My eyes never feel irritated.<br>24. I think that I could be better looking.<br>25. I am never bothered by my vision correction when I am active (sports, dance, etc.).<br>26. My vision correction is sometimes hard to put on or take off.<br>27. My friends only say good things about my vision correction.<br>28. I wish I had a different kind of vision correction.<br>29. My vision is always excellent<br>30. I am sometimes uncomfortable when I wear my vision correction.<br>31. When I wear my vision correction, I like how I look.<br>32. I am worse at sports because my vision correction bothers me.<br>33. My vision correction never gets lost or broken.<br>34. My friends sometimes say things that are not nice about my vision correction.<br>35. I like to wear my vision correction.<br>36. Sometimes my vision is not clear.<br>37. My eyes always feel great.<br>38. When I look in the mirror, I do not like how I look.<br>39. I never have any problems when I wear my vision correction while I play sports or do other activities.	100. Strongly disagree<br>75. Disagree<br>50. Neutral<br>25. Agree<br>0. Strongly agree

Instruments	Source	List of domains/subscales	List of Items	Rating scales
			40. Sometimes it is hard to clean my vision correction.<br>41. My friends never mention my vision correction.<br>42. In general, wearing my vision correction bothers me.<br>43. I can always see well than my friends.<br>44. Sometimes I don't like how my eyes feel.<br>45. Nobody notices when I wear my vision correction.<br>46. I could be better at sports if I didn't have to wear vision correction.<br>47. It is easy to put on or take off my vision correction.<br>48. My friends sometimes laugh about my vision correction.<br>49. I don't even notice my vision correction.<br>50. My friends usually see well than me.<br>51. Wearing my vision correction is always comfortable.<br>52. Wearing my vision correction makes me look worse.<br>53. I can play outside without ever thinking about my vision correction.<br>54. I don't like cleaning my vision correction.<br>55. When I wear my vision correction, my friends like the way I look.<br>56. I hate wearing vision correction.	
(SREEQ)	Crescioni M et al, 2014	Unidimensional scale measuring the impact of uncorrected and corrected refractive error on visual-related quality of life.	1. When I wear my glasses, I have problems seeing clearly.<br>2. When I wear my glasses, my vision is very clear<br>3. When I wear my glasses, my vision is blurry.<br>4. When I wear my glasses, I have to squint to see things clearly.<br>5. When I wear my glasses, I have problems seeing the computer or video games clearly.<br>6. When I wear my glasses, I have problems reading.<br>7. When I wear my glasses, I can see clearly at far.<br>8. When I wear my glasses, I have problems seeing the board, at the movies, or other things far away.<br>9. When I wear my glasses, I have problems recognizing people across the street or down the hall.<br>10. When I wear my glasses, I get headaches, or my head or eyes hurt when I read<br>11. When I wear my glasses, my nose or ears hurt<br>12. When I wear my glasses, things look distorted, slanted or double.<br>13. When I wear my glasses, I feel dizzy. 14. I have a problem wearing my glasses when I play outdoors<br>15. I am bothered by my glasses when I play sports, dance or do other activities. 16. When I wear my glasses, I do better on tests.<br>17. When I wear my glasses, my classmates make fun of me<br>18. When I wear my glasses, I like how I look<br>19. I like my frames<br>20. If I didn't wear glasses, I would look better<br>21. When I wear my glasses, my schoolwork is easier	3-point frequency scale.<br>1. All of the time<br>2. Some/most of the time<br>3. None of the time

Instruments	Source	List of domains/subscales	List of Items	Rating scales
			22. When I wear my glasses, my friends like the way I look<br>23. When I wear my glasses, my family members like the way I look	

Total number of Items generated from 20 instruments: 852.

How many subscales: 16

<u>SA1: The pool of Items generated from the Literature review.</u>

Items Because of your eyesight and with your glasses or low vision aids if you use them how difficult do you find…

1. Your math lessons?
2. Your science lessons?
3. Your geography lessons?
4. Your language lessons?
5. Reading textbooks and worksheets that are given in your school?
6. Reading the smallest print in your textbook?
7. Drawing, coloring, or painting?
8. Reading text messages on your mobile phone?
9. Reading restaurant menus?
10. Reading the board in your classroom?
11. Watching television?
12. Watching a film at the cinema?
13. Going out alone in the daylight?
14. Walking in a crowded place?
15. Using public transport (bus/train)?
16. Reading bus/train timetable on a screen at a station?
17. Chatting with your friends?
18. Recognizing faces or identifying your friends at arm's length?
19. Seeing your friend in a playground?
20. Playing video games e.g., a PlayStation?
21. Playing computer games?
22. Using IPOD/ MP3/MP4 to listen to music? Subscale 7: Sports
23. Swimming?

24. Taking part in athletics?
25. Playing ball games?

26. Do your eyes make it hard to learn?
27. Do you have a hard time seeing?
28. Do you have to do things differently than other people because of your eyes?
29. Do your eyes make it hard to concentrate?
30. Do your eyes make it hard to do certain things?
31. Do you have trouble reading close-ups?
32. Do you have to do certain things to help you see well?
33. Is it hard to see the board at school?
34. Do you run into things because of your eyes?
35. Do your eyes get tired easily?
36. Does it bother you because your eyes make it hard to learn?
37. Does it bother you because your eyes make it hard to play sports?
38. Does it bother you because you have a hard time seeing?
39. Does it bother you that you can't do certain things because of your eyes?
40. Does it bother you because your eyes make it hard to do certain things?
41. Does it bother you because it's hard to see the board at school?
42. Does it bother you because it's hard to see steps when you walk?
43. Does it bother you that bright light makes it hard to do things outside?
44. Is taking care of your eye condition hard for you?
45. Does it bother you because your eyes hurt?
46. Does it bother you that it's hard to play/interact with others because of your eyes?
47. Does your eye condition cause problems in your family?
48. Are you shy because of your eyes?
49. Do other people get frustrated with you because of your eyes?
50. Do you get teased because of your eyes?
51. Do you worry about getting hurt because of your eyes?
52. Do you worry about your eyes getting worse?
53. Do you worry about what other people think about you because of your eyes?
54. Do you worry about getting teased because of your eyes?
55. Do you worry about your eyes?
56. Are you bothered by the things you have to do to make your eyes better?
57. Do your eyes make you feel unsure of yourself?
58. Are you frustrated because your eyes aren't getting better?

59. Does it bother you when other people say things or ask questions about your eyes?
60. Do you feel “different” because of your eyes?
61. Does it bother you that you get extra attention because of your eyes?
62. Do you feel left out because of your eyes?
63. Do you worry that your eyes will make it hard to do things when you’re older?
64. Do you worry about not being able to do things because of your eyes?
65. Do you worry about what you might have to do to make your eyes better?
66. In general, would you say that your child’s overall health is: [Q]
67. At present, would you say that your child’s eyesight using both eyes is? [Q]
68. If your child has an eyesight problem for only one eye, would you say that your child’s eyesight in the affected eye is: [Q] [NA]
69. How much of the time do you worry about your child’s eyesight? [F]
70. How much time do you need to spend on treatment for your child’s vision problem (eye doctor appointments, patching, eye drops, and therapy)? [F]
71. Does the time you spend on your child’s vision problem (eye doctor appointments, patching, eye drops, and therapy) take away from time you would like to spend with your other children or husband/wife? [F]
72. Do you and other family members (your spouse or parents) argue about the medical care your child is getting or about the treatment that the doctor has prescribed? [F]
73. I am afraid that my child will never have good vision. [A] [NA]
74. I am bothered by other people’s comments about my child’s vision or eyes when I take him/her to a store or mall. [A] [NA]
75. My child likes to try new things. [A] [NA]
76. Taking my child to the eye doctor is stressful. [A] [NA]
77. I think that my child’s vision will improve. [A] [NA]
78. My child feels different from other children. [A] [NA]
79. My child is happy most of the time. [A] [NA]
80. I notice other children looking at my child. [A] [NA]
81. My child likes to visit with relatives. [A] [NA]
82. My child is teased because of his/her vision problems. [A] [NA]
83. My child cries a lot. [A] [NA]
84. I worry that my child may not be able to read, watch TV, or drive a car. [A] [NA]
85. My child makes new friends easily. [A] [NA]
86. My child is affectionate. [A] [NA]
87. My child gets along well with our other children and friends. [A] [NA]
88. My child gets angry or frustrated because of his vision problem. [A] [NA]
89. We stay at home a lot because of my child’s vision problem. [A] [NA]
90. My child can feed himself/herself. [D] [TY]
91. My child plays with toys. [D] [TY]

92. My child can recognize faces (friends, relatives) across a room. [D] [TY]
93. My child can imitate others (make a face, stick tongue out, play peek-a-boo). [D] [TY]
94. My child can dress himself/herself. [D] [TY]
95. My child can brush his/her teeth. [D] [TY]
96. My child can wash his/her face. [D] [TY]
97. My child adjusts to changes in lighting (going out into bright sunlight or entering a dark room or theater.) [D] [TY]
98. My child can ride a bicycle. [D] [TY]
99. My child can play a sport or active game (for example, tag). [D] [TY]
100. My child will track a mobile or a moving toy. [D] [TY]
101. My child can locate a small piece of food (a raisin or Cheerio) and grasp it. [D] [TY]
102. My child can pour liquid into a cup or glass. [D] [TY]
103. My child can dial a telephone. [D] [TY]
104. My child helps with chores. [D] [TY]
105. My child can tell what time it is. [D] [TY]
106. My child can identify coins. [D] [TY]
107. My child enjoys looking at books. [A] [TY]
108. My child is interested in playing with our pet(s). [A] [TY]
109. My child has a regular sleep routine. [A] [TY]
110. My child's eyesight makes it difficult for him/her to learn to walk, run, skip, or jump. [A] [TY]
111. My child's vision gets in the way of his/her learning. [A] [TY]
112. My child's eyesight has made it difficult for him/her to learn to read. [A] [TY]
113. My child enjoys watching television, videos, or playing video games. [A] [TY]
114. My child likes to travel on family vacations. [A] [TY]
115. My child enjoys playing with others (sisters and brothers or friends). [A] [TY]
116. My child enjoys drawing, painting or other art activities. [A] [TY]
117. My child's eyesight makes it difficult for him/her to find something on a crowded shelf or in a closet. [A] [TY]
118. My child makes eye contact with me and smiles. [F] [TY]
119. My child bumps into people, walls, or furniture. [F][TY]
120. My child trips over curbs or steps. [F] [TY]
121. My child bumps into other people. [F] [TY]
122. I have trouble applying treatment (for example, putting on an eye patch or glasses, giving eye drops or other medication). [F]
123. My child is uncomfortable when treated (for example, while wearing a patch or glasses or when you put in eye drops). [F]
124. My child is less active when treated (for example, when wearing a patch or glasses, or when taking eye drops or medication). [F]
125. I worry when my child refuses treatment (for example, pulls off the patch or glasses, or squeezes eye shut when trying to put in

eye drops). [F]
126. I sometimes forget to treat my child. [F]
127. Do you have any difficulty in making out whether the person you are seeing across the read is a boy or a girl, during the day?
228. Do you have any difficulty in seeing whether somebody is calling you by waving his or her hand from across the road?
129. Do you have difficulty walking alone in the corridor at school without bumping into objects or people?
130. Do you have any difficulty walking home at night (from tuition or a friend's house) without assistance when there are streetlights?
131. Do you have any difficulty copying from the blackboard while sitting on the first bench in your class?
132. Do you have difficulty in reading the bus numbers?
133. Do you have any difficulty in reading the other details on the bus (such as its destination?)
134. Do you have any difficulty in reading your textbooks at arm's length?
135. Do you have any difficulty in writing along a straight line?
136. Do you have any difficulty in finding the next line while reading when you take a break and then resume reading?
137. Do you have any difficulty in locating dropped objects (pen, pencil, and eraser) within the classroom?
138. Do you have any difficulty in threading a needle?
140. How much difficulty do you have in distinguishing between 1 rupee and 2 rupee coins (without touching)?
141. Do you have difficulty in climbing up or down stairs?
142. Do you have difficulty in lacing your shoes?
143. Do have difficulty locating a ball while playing in the daylight?
144. Do you have difficulty applying the paste on your toothbrush?
145. Do you have difficulty locating food on your plate while eating?
146. Do you have difficulty in identifying colors (e.g., while coloring)?
150. How do you think your vision is compared with that of your normal-sighted friend? Do you think your vision is?

During the Last Two Weeks, Because of Conjunctivitis...
151. Did you feel burning in your eyes?
152. You had trouble staying in air-conditioned rooms?
153. You had to use tissues
154. You had puffy eyes
155. You had problems in the light
156. You had to tear
157. You had itchy eyes
158. You had red eyes
159. You had blurred vision
160. You had eye secretion
161. You had to use eyedrops.

162. You had, in the morning, closed and sticky eyes
163. You had trouble playing outdoors
164. You had trouble practicing sports (football, gym)
165. You had trouble meeting your friends
#you had trouble:
166. Going to the swimming pool
167. Watching TV
168. Playing video and computer games
169. Playing other games, e.g., board games or card games
170. Using the computer for homework
171. Reading food packets, labels, or recipes
172. Doing household chores, e.g., washing up
173. Telling the time on a wristwatch
174. Telling the time on a wall clock
175. Using the computer for lessons
176. Reading small print textbooks, worksheets, and exam papers
177. Reading enlarged textbooks, worksheets, and exam papers
178. Drawing or painting
179. Reading handwriting
180. Seeing the board in the class
181. Recognizing people, e.g., in school corridors
182. Recognizing other people’s facial expressions
183. Finding friends in the playground
184. Taking part in science classes
185. Taking part in geography classes
186. Taking part in math classes
187. Taking part in Physical Education
188. Taking part in English classes
189. Keeping up with the teacher in lessons
190. Keeping up with other students in class
191. Getting around the school by yourself
192. Getting around outdoors by yourself
193. Reading signs and posters at stations or shops
194. Getting around in crowds by yourself
195. Seeing small moving objects, e.g., balls
196. Seeing large moving objects, e.g., cars passing Using

197. Using the escalators
198. Playing team sports, e.g., football, without adaptations
199. Watching films in the cinema
200. Watching plays and shows in the theatre
201 Reading price tags
202. Finding the correct money to pay when I am having dinner with my family
203. Can you kick a ball when you play?
204. Do you worry about your child's eyesight?
205. Can you find food on your plate?
206. My child can locate a small piece of food and grasp it when eating.
207. Can you see the bus numbers?
208. My child can recognize faces (friends, relatives) across
209. Usually, how happy do you feel?
210. How much do you think that you are good-looking?
211. How much do you feel that other children want to be your friend?
212. If you have a sister or brother, how much do you enjoy playing with them?
213. Do you have many friends?
214. How easy is it for you to make new friends?
215. How clearly (well) can you see the picture on your TV?
216. How clearly (well) can you see the moving picture in hand-held computer games?
217. How clearly (well) can you see pictures in a book?
218. How easy is it for you to put the pen cap back on?
219. How easy is it for you to cut shapes with scissors?
220. How easy is it for you to draw a straight line with a ruler?
221. How good are you at writing exactly on the line?
222. How easy is it for you to see steps when you walk up or down stairs?
223. How often do you do the right thing?
224. How much do you enjoy reading the smallest print in your textbook?
225. How easy is it for you to draw, colour in a picture, or write words at school?
226. How clearly (well) can you see the smallest writing on the board at school?
227. When you read a story, how easy is it for you to explain it to someone else?
228. How clever do you think you are?
229. How easy is it for you to answer the questions in the class at school?
230. Watching TV
231. Playing video and computer games
232. Playing indoor games, such as board games or card games

233. Using the computer at home to do my homework
234. Reading food packets, tickets, labels, or recipes
235. Doing household chores, for example, washing up or tidying my bedroom
236. Looking after my appearance, for example, doing my hair, shaving, or putting on make-up
237. Making myself a snack at home
238. Making myself a meal
239. Finding objects, I have dropped such as coins or glasses on a low-contrast surface
240. Using the computer at school or college to do schoolwork/ coursework.
241. Reading small print textbooks, worksheets, and exam papers
242. Reading other people's handwriting
243. Seeing the board in the classroom when sitting at the front
244. Recognizing people, for example, in corridors at school/college or shops
245. Recognizing other people's facial expressions when they are close to me/at arm's length
246. Finding friends in crowded areas
247. Doing math's
248. Doing science
249. Doing sports at school/college
250. Keeping up with the teacher or tutor in lessons
251. Keeping up with other students in lessons
252. Getting around school/college by myself
253. Playing team sports, such as football, without adaptations such as special balls
254. Seeing small balls when playing games, such as tennis or cricket
255. Seeing big moving objects, such as bikes passing, in daylight
256. Getting around outdoors e.g., shops or the park, by myself when it's daylight
257. Getting around outdoors e.g., shops or the park, by myself when it's dark
258. Getting around in crowds by myself
259. Finding my way around an unfamiliar house or a new building
260. Reading signs and posters at stations or shops
261. Finding the correct money to pay when shopping
262. Watching films in the cinema
263. Watching shows, such as plays at the theatre
264. Crossing the road by myself
265. Using public transport, such as trains, buses, or the tube by myself
266. Using a mobile phone to text people
267. Using a mobile phone or tablet for social networking, for example, Facebook, Twitter or Myspace
268. Reading enlarged worksheets and textbooks like dictionaries

269. Drawing or painting
270. Playing outdoor games, such as tag or hide-and-seek

***Final questionnaire in the age group of 5 to 9 years:**
271. Does your child have any difficulty walking alone in the corridor at school without bumping into objects or people?
272. Does your child have any difficulty in copying from the blackboard while sitting on the front bench in your class?
273. Does your child have any difficulty in reading the bus number?
274. Does your child have any difficulty in reading your textbooks at arm's length?
275. Does your child have any difficulty in writing along a straight line?
276. Does your child have any difficulty in finding the next line while reading when you take a break and then resume reading?
277. Does your child have any in locating the dropped objects within the classroom?
278. Does your child have any difficulty in locating/identifying food on your plate while eating?
279. Because of vision how much problem does your child have in seeing if there are animals or vehicles when walking?
280. Because of vision how much problem does your child have difficulty in going out at night?
281. Because of vision how much problem does your child have difficulty in locking or unlocking the door?
282. Because of vision how much problem does your child have difficulty in seeing differences in colors?
283. Because of vision how much problem does your child have difficulty in going to social functions such as weddings/birthday parties?
284. Because of vision how much problem does your child have in finding your way indoors?
285. Because of an eye problem do you feel frightened that your child may lose remaining vision?
286. Does your child have reduced vision?

***In the age group of 10 to 15 years**:
287. Because of your vision, how much problem do you have in finding your way indoors
288. Because of your vision, how much problem do you have in seeing differences in colors
289. Do you have difficulty in applying toothpaste on your toothbrush?
290. Because of your vision, how much problem do you have in making out differences in coins or notes
291. Do you have any difficulty in climbing up and down the stairs?
292. Because of your vision, how much problem do you have in going to the toilet
293. Do you have any difficulty in locating/identifying food on your plate while eating?
294. Do you have difficulty in lacing your shoe?
295. Because of your vision, how much problem do you have in seeing the steps of the bus when climbing in or out
296. Because of your vision, how much problem do you have in seeing if there are animals or vehicles when walking?
297. Because of your vision, how much problem do you have in going to social functions such as weddings/birthday parties
298. Because of your vision, how many problems do you have in going out at night
299. Do you have difficulty locating a ball while playing with it in daylight?

300. Because of your vision how much problem do you have in recognizing the face of a person standing near you
301. Do you have any difficulty in finding the next line while reading when you take a break and then resume reading?
302. Do you have any difficulty in writing along a straight line?
303. Do you have any difficulty in reading your textbooks at arm’s length?
304. Because of your vision, how much problem do you have in seeing when coming into the house after being in the sunlight
305. Because of your eye problem, you are ashamed that you can't see
306. Because of your vision, how much problem do you have in seeing outside in bright sunlight
307. Because of your vision, how much problem do you have in recognizing people from a distance
308. Do you have any difficulty in reading the bus number?
309. Do you have any difficulty copying from the blackboard while sitting on the front bench in your class?
310. How do you think your vision is compared with that of your normal-sighted friend?
311. Do you have reduced vision
312. Because of your eye problem do you feel frightened that you may lose your remaining vision
313. Because of your eye problem do you feel you have become a burden on others?

# Do you have difficulty in....?
314. Walking without bumping into objects/people
315. Getting around alone in places you know at night time
316. Reading the bus numbers
317. Finding out the next line while reading
318. Locating dropped objects
319. Threading a needle
320. Locating ball while playing in the daytime
321. Copying from your friend’s notebook
322. Reading the rates on items (e.g., chips packets, biscuit packets)
323. Reading the phone numbers on mobile phone
324. Watching TV
325. Seeing time in your wristwatch
326. Moving around safely in places you don’t know in the daytime
327. Moving around safely in places you don’t know at night-time
328. Reading from a computer screen
329. Copying the small letters from the board
330. Walking on uneven ground
331. Reading shop names
332. Seeing animals in zoo
333. Identifying dirt, stains on your own clothes

334. Reading your books at near
335. Seeing your friend in the playground while playing
336. Watching a movie at the theatre
337. Selecting a song using iPod
338. Playing video games
339. Seeing the numbers and markings on the scale

# how do you think about......?

340. Your vision compared to your normally sighted friend?

341. Are you confident that you can move around safely in places you don't know at night-time?
342. Are you confident in places you don't know?
343. Do you get frustrated?
344. When you ask for help, do people understand how much help you need?
345. Are you confident to use public transport (such as buses, trains, ferries) by yourself?
346. Can you find your friends in the playground at lunch and play time?
347. Do other students understand your special needs?
348. Do people tell you that you can't do the things that you want to do?
349. Are you confident that you can move around safely in places you don't know in the daytime?
350. When you are in a room, can you recognize people you know before they speak to you?
351. Has your eyesight stopped you from doing the things that you want to do?
352. Do other students help you to join in with them?
353. Do you find it hard to join in with other students?
354. Do you get the chance to go to activities other than sports (such as social groups)?
355. Do you get all the information at the same time as the other students?
356. Do you get enough time in school to complete the work set by the teacher?
357. Can you take part in games or sports that you want to play with your friends?
358. Are you confident to make your way to school?
359. Do other students help you when you ask them for help?
360. When you are in the classroom, are you confident about asking for the help you need?
361. Do people stop you from doing the things you want to do?
362. Do you find it difficult to go downstairs or to step off the footpath?
363. In the classroom, do you get all the same information as other students?
364. Do your teachers understand your special needs?

How often are these tasks hard for you to do?
(With glasses or contacts, if you wear them)

365. It is hard to see the board if I am sitting in the back of the room.

366. It is hard to see the pictures on the television screen from across the room.
367. It is hard to see someone’s facial expressions (smiling, frowning) when they are talking to me.
368. It is hard to see someone’s face from across the room.
369. It is hard to see the words in my books if I am holding my book far from my face.
370. It is hard to see the steps, so I do not trip when going up the stairs.
371. It is hard to see how much to fill a glass with a drink.
372. It is hard to see where to write on lined notebook paper.
373. It is hard to adjust to see where to sit when I walk into a dark room.
374. It is hard to adjust to see when I go from a dark room to a brightly lit room.
375. It is hard to see when I first walk outside into the sunlight.
376. It is hard to play sports that use small balls like baseball, tennis, or golf.
377. It is hard to see the cursor on the computer screen.
378. It is hard to see words on a tablet or computer if I do not make the font larger.
379. It is hard to see text messages on a cell phone.
380. It is hard to do activities because of the pain in my eyes.

How often is this true for you?
381. I feel left out of activities because of my vision.
382. I do not join activities with friends because of my vision.
383. I do not like using eye drops.
384. I let my eye disease stop me from doing what I want to do.
385. I do not like getting injections or infusions for my eye disease.
386. I do not like having others know about my eye disease.
387. I get frustrated because I cannot do things because of my vision.
388. I do not like the way my eyes look.
389. I use devices or special aids to help me see, such as:
A. large print books, cards, or games?
B. Magnifying glass?
C. Special lamps or lights?
D. Other (not including glasses or contacts):
390. I do not like using devices or special aids to help me see (not including glasses or contacts).
391. I wear my glasses or contacts like I am
supposed to.
392. I do not like to wear my glasses or contacts.

393. At present, my eyesight using both eyes is (with glasses or contacts, if you wear them):

394. When I wear my glasses, I have problems seeing clearly.
395. When I wear my glasses, my vision is very clear
396. When I wear my glasses, my vision is blurry.
397. When I wear my glasses, I have to squint to see things clearly.
398. When I wear my glasses, I have problems seeing the computer or video games clearly.
399. When I wear my glasses, I have problems reading.
400. When I wear my glasses, I can see clearly at far.
401. When I wear my glasses, I have problems seeing the board, at the movies, or other things far away.
402. When I wear my glasses, I have problems recognizing people across the street or down the hall.
403. When I wear my glasses, I get headaches, or my head or eyes hurt when I read
404. When I wear my glasses, my nose or ears hurt
405. When I wear my glasses, things look distorted, slanted or double.
406. When I wear my glasses, I feel dizzy. 14. I have a problem wearing my glasses when I play outdoors
407. I am bothered by my glasses when I play sports, dance or do other activities. 16. When I wear my glasses, I do better on tests.
408. When I wear my glasses, my classmates make fun of me
409. When I wear my glasses, I like how I look
410. I like my frames
411. If I didn't wear glasses, I would look better
412. When I wear my glasses, my schoolwork is easier
413. When I wear my glasses, my friends like the way I look
414. When I wear my glasses, my family members like the way I look
415. My vision is very clear when I look far away (movies or board at school).
416. My eyes are sometimes uncomfortable.
417. I am happy with the way that I look.
418. When I play sports or other activities, I sometimes don't wear vision correction because it bothers me.
419. When I play outdoors, I never have a problem with my vision correction.
420. My friends make fun of me because of my vision correction.
421. I love my vision correction.
422. When I look far away, my vision is not as clear as I would like it to be.
423. My eyes are always comfortable.
424. I do not like how I look when I wear my vision correction.
425. When I play outdoors, I never have a problem with my vision correction.
426. My vision correction sometimes breaks or falls off while I am wearing it.
427. My friends want the same kind of vision correction that I have.
428. I don't like my vision correction very
Much

429. My vision is very clear when I look at something close (books or cell phones).
430. My eyes sometimes itch, burn, or feel.
431. My vision correction makes me look cool.
432. When I play outside, my vision correction sometimes bothers me.
433. When I am active, my vision correction never falls off.
434. My friends don’t like how I look when I wear my vision correction.
435. I never have problems with my vision correction.
436. When I read, my vision is not as clear as I would like it to be.
437. My eyes never feel irritated.
438. I think that I could be better looking.
439 I am never bothered by my vision correction when I am active (sports, dance, etc.).
440. My vision correction is sometimes hard to put on or take off.
441. My friends only say good things about my vision correction.
442. I wish I had a different kind of vision correction.
443. My vision is always excellent
444. I am sometimes uncomfortable when I wear my vision correction.
445. When I wear my vision correction, I like how I look.
446. I am worse at sports because my vision correction bothers me.
447. My vision correction never gets lost or broken.
448. My friends sometimes say things that are not nice about my vision correction.
449. I like to wear my vision correction.
450. Sometimes my vision is not clear.
451. My eyes always feel great.
452. When I look in the mirror, I do not like how I look.
453. I never have any problems when I wear my vision correction while I play sports or do other activities.
454. Sometimes it is hard to clean my vision correction.
455. My friends never mention my vision correction.
456. In general, wearing my vision correction bothers me.
457. I can always see well than my friends.
458. Sometimes I don’t like how my eyes feel.
459. Nobody notices when I wear my vision correction.
460. I could be better at sports if I didn’t have to wear vision correction.
461. It is easy to put on or take off my vision correction.
462. I don’t even notice my vision correction.
463. I hate wearing vision correction
464. I worry about my eyes

465. It bothers me that people wonder what is wrong with my eyes
466. It bothers me because I have to wait for my eyes to clear up
467. Kids tease me because of my eyes
468. I am bothered when grownups say things about my eyes
469. I am bothered when my parents say things about my eyes
470. It bothers me that I have to shut one eye when it is sunny
471. I feel different from other kids because my eyes go in and out
472. I worry about what other people think of me because of my eyes
473. My eyes make it hard to look people in the eye
474. It is hard to concentrate because of my eyes
475. My eyes make it hard for me to make friends
476. I worry about my child's eyes
477. I worry that my child will be less independent because of his/her eyes
478. I worry that my child will have permanent damage to his/her eyes
479. I worry that my child doesn't see well
480. I worry about how my child's eyes will affect him/her socially
481. I worry that my child will get hurt physically because of his/her eyes
482. I worry about the possibility of surgery 8. I worry about my child becoming self-conscious because of his/her eyes.
483. I worry that my child will not be able to see the board at school
484. I worry about other kids teasing my child because of his/her eyes
485. It worries me what others will think about my child because of his/her eyes 12. I worry that my child's eye condition will affect his/her personality.
486. I worry that my child's eyes will affect his/her social life if nothing is done
487. I worry about my child's eyesight long term
489. I worry about my child's depth perception
490. I worry about whether or not my child should have surgery
491. I worry about my child's ability to make friends.

492. Do your eyes feel tired when reading or doing close work?
493. Do your eyes feel uncomfortable when reading or doing close work?
494. Do you have headaches when reading or doing close work?
495. Do you feel sleepy when reading or doing close work?
496. Do you lose concentration when reading or doing close work?
497. Do you have trouble remembering what you have read?
498. Do you have double vision when reading or doing close work?
499. Do you see the words move, jump, swim, or appear to float on the page when reading or doing close work?

500. Do you feel like you read slowly?
501. Do your eyes ever hurt when reading or doing close work?
502. Do your eyes ever feel sore when reading or doing close work?
503. Do you feel a “pulling” feeling around your eyes when reading or doing close work?
504. Do you notice the words blurring or coming in and out of focus when reading or doing close work?
505. Do you lose your place while reading or doing close work?
506. Do you have to re-read the same line of words when reading?

507. Are you feeling sad due to the patch?
508. Feeling of your patch on your face (like sticky, or itchy)
509. Are you hurt as a result of the patch?
510. Does the patch affect you when you’re doing work at school (like reading and writing?)
511. Cross
512. How other children have treated you (like laughing at you, or calling you names) because of your patch
513. Does the patch affect you when doing things (like playing on the computer, colouring, playing games, watching TV)
514. Are you worried about the results of the patch?
515. Are you upset about the results of the patch?
516. Does the patch affect your play with my friends?
517. Does the patch affect your happiness?
# patching questionnaire
518. My child does not seem to mind wearing the patch once it is on.
519. I worry that by wearing the patch, my child may miss out on fun activities (such as games and parties).
520. Wearing patch the patch affects my child’s learning

521. Wearing the patch makes it hard for my child to play outside, such as running, jumping, or riding a bike or tricycle.
522. I have trouble putting on my child’s patch and keeping it on.
523. Wearing a patch is a source of tension or conflict in my relationship:
A. with my child.
B. with my child’s babysitter or teacher.
524. Wearing the patch makes it difficult for my child to draw, color, or write.
525. I worry that my child will become injured when wearing the patch.
526. My child can see well when wearing the patch.
527. My child complains when it is time to wear the patch.
528. Wearing the patch makes my child’s eyes or eyelids red or irritated.
529. I worry that my child does not wear the patch enough.
530. My child is clumsier and more uncoordinated than usual when wearing the patch.

531. I notice that other children stare at my child when the patch is on.
532. I believe that wearing a patch will improve my child's vision.
533. Wearing the patch makes it difficult for my child to play with blocks or toys.
534. I sometimes forget to put the patch on my child.
535. I worry that wearing the patch will make my child feel different from other children.

# Atropine questionnaire

536. My child does not seem to mind using the drops.
537. I worry that by using drops, my child may miss out on fun activities (such as games and parties).
538. Using the drops affects my child's learning
539. Using the drops makes it hard for my child to play outside such as running, jumping, or riding a bike or tricycle.
540. I have trouble putting the drops in my child's eye.
541. Using the drops is a source of tension or conflict in my relationship:
a. with my child.
b. with my child's babysitter or teacher.
542. Using the drops makes it difficult for my child to draw, colour, or write.
543. I worry that my child will become injured when using the drops.
544. My child can see well when using drops.
545. My child complains when it is time to put in the drops.
546. Using the drops makes my child's eyes or eyelids red or irritated.
547. I worry that my child does not get the drops often enough.
548. My child is clumsier and more uncoordinated than usual when using the drops.
549. I notice that other children stare at my child when the drops are in.
550. I believe that using the drops will improve my child's vision.
551. Using the drops makes it difficult for my child to play with blocks or toys.
552. I sometimes forget to put the drops in my child.
553. I worry that using the drops will make my child feel different from other children.

554. Does your child has any difficulty in walking alone in the corridor at school without bumping into objects or people
555. Does your child has any difficulty in copying from the blackboard while sitting on front bench in your class?
556. Does your child have any difficulty in reading the bus number?
557. Does your child have any difficulty in reading your textbooks at arm's length?
558. Does your child have any difficulty in writing along a straight line?
559. Does your child have any difficulty in finding the next line while reading when you take a break and then resume reading?
560. Does your child have any in locating the dropped objects within the classroom?
561. Does your child have any difficulty in locating/identifying food on your plate while eating?
562. Because of vision how much problem does your child have in seeing if there are animals or vehicles when walking?

563. Because of vision how much problem does your child have in going out at night?
564. Because of vision how much problem does your child have in locking or unlocking the door?
565. Because of vision how much problem does your child have in seeing differences in colours?
566. Because of vision how much problem does your child have in going to social functions such as weddings/birthday parties?
567. Because of vision how much problem does your child have in finding their way indoors?
568. Because of eye problems do you feel frightened that your child may lose remaining vision?
569. Does your child have reduced vision?

570. Because of your vision, how much problem do you have in finding your way indoors?
571. Because of your vision, how much problem do you have in seeing differences in colours?
572. Do you have difficulty in applying toothpaste on your toothbrush?
573. Because of your vision, how much problem do you have in making out differences in coins or notes?
574. Do you have any difficulty in climbing up and down the stairs?
575. Because of your vision, how much problem do you have in going to the toilet?
576. Do you have any difficulty in locating/identifying food on your plate while eating?
577. Do you have difficulty in lacing your shoe?
578. Because of your vision, how much problem do you have in seeing the steps of the bus when climbing in or out?
579. Because of your vision, how much problem do you have in seeing if there are animals or vehicles when walking?
580. Because of your vision, how much problem do you have in going to social functions such as weddings/birthday parties?
581. Because of your vision, how much problem do you have in going out at night?
582. Do you have difficulty in locating a ball while playing with it in daylight?
583. Because of your vision, how much problem do you have in recognizing the face of a person standing near you?
584. Do you have any difficulty in finding the next line while reading when you take a break and then resume reading?
585. Do you have any difficulty in writing along a straight line?
586. Do you have any difficulty in reading your textbooks at arm’s length?
587. Because of your vision, how much problem do you have in seeing when coming into the house after being in the sunlight?
588. Because of your eye problem, you are ashamed that you can't see.
589. Because of your vision, how much problem do you have in seeing outside in bright sunlight?
590. Because of your vision, how much problem do you have in recognizing people from a distance?
591. Do you have any difficulty in reading the bus number?
592. Do you have any difficulty copying from the blackboard while sitting on the front bench in your class?
593. How do you think your vision is compared with that of your normal-sighted friend?
594. Do you have reduced vision?
595. Because of your eye problem do you feel frightened that you may lose your remaining vision?
596. Because of your eye problem do you feel you have become a burden on others?
597. Watching TV

598. Playing video and computer games
599. Playing indoor games, such as board games or card games
600. Using the computer at home to do my homework
601. Reading food packets, tickets, labels or recipes
602. Doing household chores, for example, washing up or tidying my bedroom
603. Looking after my appearance, for example, doing my hair, shaving, or putting on make-up
604. Making myself a snack at home
605. Making myself a meal
606. Finding objects, I have dropped such as coins or glasses on a low contrast surface
607. Using the computer at school or college to do schoolwork/ coursework
608. Reading small print textbooks, worksheets, and exam papers
609. Reading other people's handwriting
610. Seeing the board in the classroom when sitting at the front
611. Recognising people, for example, in corridors at school/college or shops
612. Recognising other people's facial expressions when they are close to me/at arm's length
613. Finding friends in crowded areas
614. Doing maths
615. Doing science
616. Doing sports at school/college
617. Keeping up with the teacher or tutor in lessons
618. Keeping up with other students in lessons
619. Getting around school/college by myself
620. Playing team sports, such as football, without adaptations such as special balls
621. Seeing small balls when playing games, such as tennis or cricket
622. Seeing big moving objects, such as bikes passing, in daylight
623. Getting around outdoors e.g., shops or the park, by myself when it's daylight
624. Getting around outdoors e.g., shops or the park, by myself when it's dark
625. Getting around in crowds by myself
626. Finding my way around an unfamiliar house or a new building
627. Reading signs and posters at stations or shops
628. Finding the correct money to pay when shopping
629. Watching films in the cinema
630. Watching shows, such as plays at the theatre
631. Crossing the road by myself
632. Using public transport, such as trains, buses, or the tube by myself
633. Using a mobile phone to text people

634. Using a mobile phone or tablet for social networking, for example, Facebook, Twitter or Myspace
635. Reading enlarged worksheets and textbooks like dictionaries
636. Drawing or painting
637. Playing outdoor games, such as tag or hide and seek.
638. In general, would you say that your child's overall health is?
639. At present, would you say that your child's eyesight using both eyes is?
640. If your child has an eyesight problem for only one eye, would you say that your child's eyesight in the affected eye is:
641. How much of the time do you worry about your child's eyesight?
642. How much time do you need to spend on treatment for your child's vision problem (eye doctor appointments, patching, eye drops, and therapy)?
643. Does the time you spend on your child's vision problem (eye doctor appointments, patching, eye drops, and therapy) take away from time you would like to spend with your other children or husband/wife?
644. Do you and other family members (your spouse or parents) argue about the medical care your child is getting or about treatment that the doctor has prescribed?
645. I am afraid that my child will never have good vision.
646. I am bothered by other people's comments about my child's vision or eyes when I take him/her to a store or mall.
647. My child likes to try new things.
648. Taking my child to the eye doctor is stressful.
649. I think that my child's vision will improve.
650. My child feels different from other children.
651. My child is happy most of the time.
652. I notice other children looking at my child.
653. My child likes to visit with relatives.
654. My child is teased because of his/her vision problems.
655. My child cries a lot.
656. I worry that my child may not be able to read, watch TV, or drive a car.
657. My child makes new friends easily.
658. My child is affectionate.
659. My child gets along well with our other children and friends.
660. My child gets angry or frustrated because of his vision problem.
661. We stay at home a lot because of my child's vision problem.
662. My child can feed himself/herself.
663. My child plays with toys.
664. My child can recognize faces (friends, relatives) across a room.
665. My child can imitate others (make a face, stick tongue out, play peek-a-book).
666. My child can dress himself/herself.

667. My child can brush his/her teeth.
668. My child can wash his/her face.
669. My child adjusts to changes in lighting (going out into bright sunlight or entering a dark room or theatre.)
670. My child can ride a bicycle.
671. My child can play a sport or active game (for example, tag).
672. My child will track a mobile or a moving toy.
673. My child can locate a small piece of food (a raisin or Cheerio) and grasp it.
674. My child can pour liquid into a cup or glass.
675. My child can dial a telephone.
676. My child helps with chores.
677. My child can tell what time it is.
678. My child can identify coins.
679. My child enjoys looking at books.
680. My child is interested in playing with our pet(s).
681. My child has a regular sleep routine.
682. My child's eyesight makes it difficult for him/her to learn to walk, run, skip, or jump.
683. My child's vision gets in the way of his/her learning.
684. My child's eyesight has made it difficult for him/her to learn to read.
685. My child enjoys watching television, videos, or playing video games.
686. My child likes to travel on family vacations.
687. My child enjoys playing with others (sisters and brothers or friends).
688. My child enjoys drawing, painting, or other art activities.
689. My child's eyesight makes it difficult for him/her to find something on a crowded shelf or in a closet.
690. My child makes eye contact with me and smiles.
691. My child bumps into people, walls, or furniture.
692. My child trips over curbs or steps.
693. My child bumps into other people.
694. I have trouble applying treatment (for example, putting on an eye patch or glasses, giving eye drops or other medication).
695. My child is uncomfortable when treated (for example, while wearing a patch or glasses or when you put in eye drops).
696. My child is less active when treated (for example, when wearing a patch or glasses, or when taking eye drops or medication).
697. I worry when my child refuses treatment (for example, pulls off the patch or glasses, or squeezes eye shut when trying to put in eye drops).
698. I sometimes forget to treat my child.
# PedEyeQ -Functional vision: Child
699. Do your eyes make it hard to learn?
700. Do you have a hard time seeing?

701. Do you have to do things differently than other people because of your eyes?
702. Do your eyes make it hard to concentrate?
703. Do your eyes make it hard to do certain things?
704. Do you have trouble reading close-up?
705. Do you have to do certain things to help you see well?
706. Is it hard to see the board at school?
708. Do you run into things because of your eyes?
709. Do your eyes get tired easily?
# PedEyeQ -Bothered by eyes / vision:
710. Does it bother you because your eyes make it hard to learn?
711. Does it bother you because your eyes make it hard to play sports?
712. Does it bother you because you have a hard time seeing?
713. Does it bother you that you can’t do certain things because of your eyes?
714. Does it bother you because your eyes make it hard to do certain things?
715. Does it bother you because it’s hard to see the board at school?
716. Does it bother you because it’s hard to see steps when you walk?
717. Does it bother you that bright light makes it hard to do things outside?
718. Is taking care of your eye condition hard for you?
719. Does it bother you because your eyes hurt?
PedEyeQ -Social:
720. Does it bother you that it’s hard to play/interact with others because of your eyes?
721. Does your eye condition cause problems in your family?
722. Are you shy because of your eyes?
723. Do other people get frustrated with you because of your eyes?
724. Do you get teased because of your eyes?
725. Do you worry about getting hurt because of your eyes?
726. Do you worry about your eyes getting worse?
727. Do you worry about what other people think about you because of your eyes?
728. Do you worry about getting teased because of your eyes?
729. Do you worry about your eyes?
# PedEyeQ -Frustration / worry:
730. Are you bothered by the things you have to do to make your eyes better?
731. Do your eyes make you feel unsure of yourself?
732. Are you frustrated because your eyes aren’t getting better?
733. Does it bother you when other people say things or ask questions about your eyes?
734. Do you feel “different” because of your eyes?

735. Does it bother you that you get extra attention because of your eyes?
736. Do you feel left out because of your eyes?
737. Do you worry that your eyes will make it hard to do things when you’re older?
738. Do you worry about not being able to do things because of your eyes?
739. Do you worry about what you might have to do to make your eyes better?

#how often are these tasks hard for you to do?
(With glasses or contacts, if you wear them)

740. It is hard to see the board if I am sitting in the back of the room.
741. It is hard to see the pictures on the television screen from across the room.
742. It is hard to see someone’s facial expressions (smiling, frowning) when they are talking to me.
743. It is hard to see someone’s face from across the room.
744. It is hard to see the words in my books if I am holding my book far from my face.
745. It is hard to see the steps, so I do not trip when going up the stairs.
746. It is hard to see how much to fill a glass with a drink.
747. It is hard to see where to write on lined notebook paper.
748. It is hard to adjust to see where to sit when I walk into a dark room.
749. It is hard to adjust to see when I go from a dark room to a brightly lit room.
750. It is hard to see when I first walk outside into the sunlight.
751. It is hard to play sports that use small balls like baseball, tennis, or golf.
752. It is hard to see the cursor on the computer screen.
753. It is hard to see words on a tablet or computer if I do not make the font larger.
754. It is hard to see text messages on a cell phone.
755. It is hard to do activities because of the pain in my eyes.

#how often is this true for you?

756. I feel left out of activities because of my vision.
757. I do not join activities with friends because of my vision.
758. I do not like using eye drops.
759. I let my eye disease stop me from doing what I want to do.
760. I do not like getting injections or infusions for my eye disease.
761. I do not like having others know about my eye disease.
762. I get frustrated because I cannot do things because of my vision.
763. I do not like the way my eyes look.
764. I use devices or special aids to help me see, such as:
A. large print books, cards, or games?
B. Magnifying glass?
C. Special lamps or lights?

D. Other (not including glasses or contacts):
765. I do not like using devices or special aids to help me see (not including glasses or contacts).
766. I wear my glasses or contacts like I am supposed to.
767. I do not like to wear my glasses or contacts.
768. At present, my eyesight using both eyes are (with glasses or contacts, if you wear them):
769. When I am having dinner with my family, I feel
770. When I go to bed at night, I feel
771. My brothers and sisters make me feel
772. When I am with my grandparents, I feel
773. When I watch television, I feel
774. When I am eating, I feel
775. When I go to the doctor's, I feel
776. When I stay in the hospital, I feel
777. When I take medicine, I feel
778. When I am sick, I feel
779. When I am not sick, I feel
780. On my birthday, I feel
781. When I play alone, I feel
782. When it is a holiday, I feel
783. When people tell me what to do, I feel
784. At school, I feel
785. When I make a drawing, I feel
786. When I move (walk, run, jump), I feel
787. When I am playing outside, I feel
788. When I play a sport, I feel
789. When I do my homework, I feel
790. When I read a book, I feel
791. When my work is marked at school, I feel
792. Can you climb up and down stairs?
793. Do you bump into things?
794. Can you kick a ball when you play?
795. Can you hit the ball when you play?
796. Can you find food on your plate when eating?
797. Can you find your favorite toy at playtime?
798. Can you pick up a red pencil from a box of pencils?
799. Can you put toothpaste on your toothbrush by yourself?

800. Can you do (tie) up your shoelaces by yourself?
801. Can you see the TV clearly?
802. Can you see a person’s face across the road?
803. Can you see the bus numbers clearly?
804. Can you see the pictures in your books clearly?
805. Can you read a book by yourself?
806. Can you draw a straight line on paper without a ruler?
807. Can you copy from the board in class?
808. Can you write the numbers from 1 to 10?
809. Can you write your name?
810. Can you remember words easily?
811. Can you finish your homework on time?
812. Can you do math?
813. Is your handwriting neat?
814. In general, would you say that your child’s overall health is?
815. At present time, would you say that your child’s eyesight (using both eyes) is?
816. If your child has an eyesight problem for only one eye, would you say that your child’s eyesight in the affected eye is:
817. My child can feed himself/herself.
818. My child can recognise faces (friends, relatives) across a room.
819. My child can dress himself/herself.
820. My child can brush his/her teeth.
821. My child can wash his/her face.
822. My child can ride a bicycle.
823. My child can play a sport or an active game (for example, football).
824. My child can locate a small piece of food (a raisin or Cheerio) and grasp it.
825. My child can pour liquid into a cup or glass.
826. My child can dial a telephone.
827. My child helps with chores.
828. My child can tell what time it is.
829. My child can identify coins.
830. My child’s eyesight makes it difficult for him/her to find something on a crowded shelf or in a closet.
831. My child bumps into people, walls, or furniture.
832. My child trips over curbs or steps.
833. My child is happy most of the time.
834. My child likes to visit with relatives.
835. My child makes friends easily.

836. My child is affectionate.
837. My child gets along well with our other children and friends.
838. My child enjoys looking at books.
839. My child’s eyesight makes it difficult for him/her to learn to walk, run, skip, or jump.
840. My child’s vision gets in the way of his/her learning.
841. My child enjoys watching television, videos, or playing video games.
842. My child likes to travel on family vacations.
843. My child enjoys playing with others (sisters and brothers or friends).
844. My child enjoys drawing, painting, and other art activities.
845. How much of the time do you worry about your child’s eyesight?
846. How much time do you need to spend on treatment for your child’s vision problem (eye doctor appointment, patching, eye drops, and therapy)?
847. Does the time you spend on your child’s vision problem (eye doctor appointments, patching, eye drops, and therapy) take away from time you would like to spend with your other children or husband/wife?
848. Do you and other family members (your spouse or parents) argue about the medical care your child is getting or about treatment that the doctor has prescribed?
849. I am bothered by other people’s comments about my child’s vision or eyes when I take him/her to a store or mall.
850. My child feels different from other children.
851. I notice other children looking at my child.
852. My child is teased because of his/her vision problem.
853. I worry that my child may not be able to read, watch TV, or drive a car.
854. I have trouble applying eye/vision treatment (for example, putting on an eye patch or glasses, giving eye drops or other medication).
855. My child is uncomfortable when treated (for example, while wearing a patch or glasses or when you put in eye drops).
856. My child is less active when having treatment (for example, when wearing a patch or glasses, or when taking eye drops or Medication).
857. I worry when my child refuses treatment (for example, pulls off the patch or glasses, or squeezes eye shut when trying to put in eye drops).
858. I forget to give my child treatment.
